# Antagonistic paralogs control a switch between growth and pathogen resistance in *C*. *elegans*

**DOI:** 10.1371/journal.ppat.1007528

**Published:** 2019-01-14

**Authors:** Kirthi C. Reddy, Tal Dror, Ryan S. Underwood, Guled A. Osman, Corrina R. Elder, Christopher A. Desjardins, Christina A. Cuomo, Michalis Barkoulas, Emily R. Troemel

**Affiliations:** 1 Division of Biological Sciences, University of California, San Diego, La Jolla, CA United States of America; 2 Department of Life Sciences, Imperial College, London, United Kingdom; 3 Infectious Disease and Microbiome Program, Broad Institute, Cambridge MA United States of America; University of Texas Southwestern Medical Center at Dallas, UNITED STATES

## Abstract

Immune genes are under intense, pathogen-induced pressure, which causes these genes to diversify over evolutionary time and become species-specific. Through a forward genetic screen we recently described a *C*. *elegans*-specific gene called *pals-22* to be a repressor of “Intracellular Pathogen Response” or IPR genes. Here we describe *pals-25*, which, like *pals-22*, is a species-specific gene of unknown biochemical function. We identified *pals-25* in a screen for suppression of *pals-22* mutant phenotypes and found that mutations in *pals-25* suppress all known phenotypes caused by mutations in *pals-22*. These phenotypes include increased IPR gene expression, thermotolerance, and immunity against natural pathogens, including *Nematocida parisii* microsporidia and the Orsay virus. Mutations in *pals-25* also reverse the reduced lifespan and slowed growth of *pals-22* mutants. Transcriptome analysis indicates that *pals-22* and *pals-25* control expression of genes induced not only by natural pathogens of the intestine, but also by natural pathogens of the epidermis. Indeed, in an independent forward genetic screen we identified *pals-22* as a repressor and *pals-25* as an activator of epidermal defense gene expression. In summary, the species-specific *pals-22* and *pals-25* genes act as a switch to regulate a program of gene expression, growth, and defense against diverse natural pathogens in *C*. *elegans*.

## Author summary

Infection by microbial pathogens imposes selective pressure on animal and plant hosts. For this reason, host immune genes tend to vary in DNA sequence over evolutionary time and become ‘species-specific’. In this work we describe a pair of species-specific genes called *pals-22/pals-25* that promote resistance against natural pathogens of the small roundworm *Caenorhabditis elegans*. This gene pair acts as an on/off switch for genes that are activated after infection and rewires the physiology of worms to either be in an immune state or a growth state. In particular, the *pals-22* gene promotes development and increases lifespan, while the *pals-25* gene promotes resistance against heat shock and natural pathogens including virus and fungal-related microbes. While the DNA sequence of *pals-22* and *pals-25* appears specific to worms, the way that these two genes affect physiology may provide insight into how organisms balance growth and immunity.

## Introduction

Evolutionarily ancient genes control core processes in diverse organisms. For example, the >500 million-year-old Hox gene cluster is required for establishing body plan polarity in animals as diverse as worms, flies and humans [1]. However, evolutionarily young genes can also play key roles in development. For example, the *Drosophila* Umbrea gene only evolved within the *Drosophila* lineage in the last 15 million years but is essential for chromosome segregation in *Drosophila melanogaster* [2]. In general, the functions of evolutionarily young genes are less well understood than the functions of evolutionarily ancient genes.

New genes can arise through gene duplication and diversification [3]. Extensive gene duplication can lead to large, expanded gene families, which appear 'species-specific' if there is significant diversification away from the ancestral gene. The function of species-specific genes can provide insight into the pressures imposed upon organisms in their recent evolutionary past. Pathogen infection imposes some of the strongest selective pressure on organisms, and accordingly, many species-specific, expanded gene families are involved in immunity. One example is the family of mouse Naip genes, which encode sensor proteins in the inflammasome that detect bacteria to trigger cytokine release and cell death [4]. Another example is the plant R genes, which detect virulence factors from co-evolved pathogens to activate effector-triggered immunity [5]. Interestingly, a growing theme in plant R genes is that they can function as opposing gene pairs, with one R gene promoting host defense and the other R gene inhibiting host defense. Of note, both the Naip and R genes were identified through unbiased forward genetic screens for immune genes.

Recently, we described a forward genetic screen in *C*. *elegans* for genes that regulate the transcriptional response to natural intracellular pathogens [6]. From this screen we identified a *C*. *elegans*-specific gene called *pals-22* that regulates expression of Intracellular Pathogen Response or IPR genes. Interestingly, we found that *pals-22* also regulates proteostasis, potentially through ubiquitin ligase activity (see below). The ‘*pals’* signature stands for protein containing ALS2CR12 signature, which is found in the single *pals* gene in humans called ALS2CR12. A genome-wide association study implicated ALS2CR12 in amyotrophic lateral sclerosis (ALS) [7], although this gene has no known role in ALS, and its biological function is unknown. The *pals* gene family has only a single member each in the mouse and human genomes but is substantially expanded in *Caenorhabditis* genomes: *C*. *elegans* has 39 *pals* genes; *C*. *remanei* has 18 *pals* genes; *C*. *brenneri* has 8 *pals* genes; and *C*. *briggsae* has 8 *pals* genes [8].

*pals-22* mutants have several striking phenotypes in *C*. *elegans*. First, *pals-22* mutants have upregulated expression of several IPR genes including the cullin gene *cul-6*, which is predicted to encode a component of a Cullin-Ring Ligase complex. Second, *pals-22* mutants have increased tolerance of proteotoxic stressors, and this increased tolerance requires the wild-type function of *cul-6*. Third, *pals-22* mutants have less robust health in the absence of stressors. In particular, they have slowed development and shorter lifespans compared to wild-type animals. Fourth, as shown by another group who identified *pals-22* in an independent forward genetic screen, *pals-22* mutants have increased transgene silencing and increased RNA interference (RNAi) against exogenous RNA [8]. Thus, loss-of-function mutations in *pals-22* appear to broadly reprogram the physiology of *C*. *elegans*.

Here we describe a forward genetic screen for suppressors of *pals-22* and identify another *pals* gene called *pals-25*. Interestingly, although it appears that *pals-25* and *pals-22* are in an operon together, these two genes function antagonistically and direct opposing phenotypes. We show that mutations in *pals-25* strongly suppress all the physiological phenotypes seen in *pals-22* mutants, including IPR gene expression, stress resistance, lifespan, development and transgene silencing. Furthermore, we find that *pals-22* mutants have increased resistance against natural intracellular pathogens, like the microsporidian species *Nematocida parisii* and the Orsay virus. This increased resistance is suppressed by mutations in *pals-25*. Also, we use RNA-seq analysis to show that the *pals-22/pals-25* gene pair (hereafter referred to as *pals-22/25*) regulate expression of a majority of the genes induced by natural pathogens of the intestine and find that most of these genes are also induced by blockade of the proteasome. Interestingly, we observe that *pals-22* and *pals-25* also regulate expression of genes induced by natural eukaryotic pathogens infecting through the epidermis. Indeed, in an independent forward genetic screen to find regulators of epidermal defense gene expression we identified additional mutant alleles of *pals-22* and *pals-25*. In summary, the species-specific *pals-22/25* gene pair control an entire physiological program that balances growth with increased proteostasis capacity and resistance against diverse natural pathogens.

## Results

### *pals-25* is required for increased IPR gene expression in *pals-22* mutants

**Fig 1:**
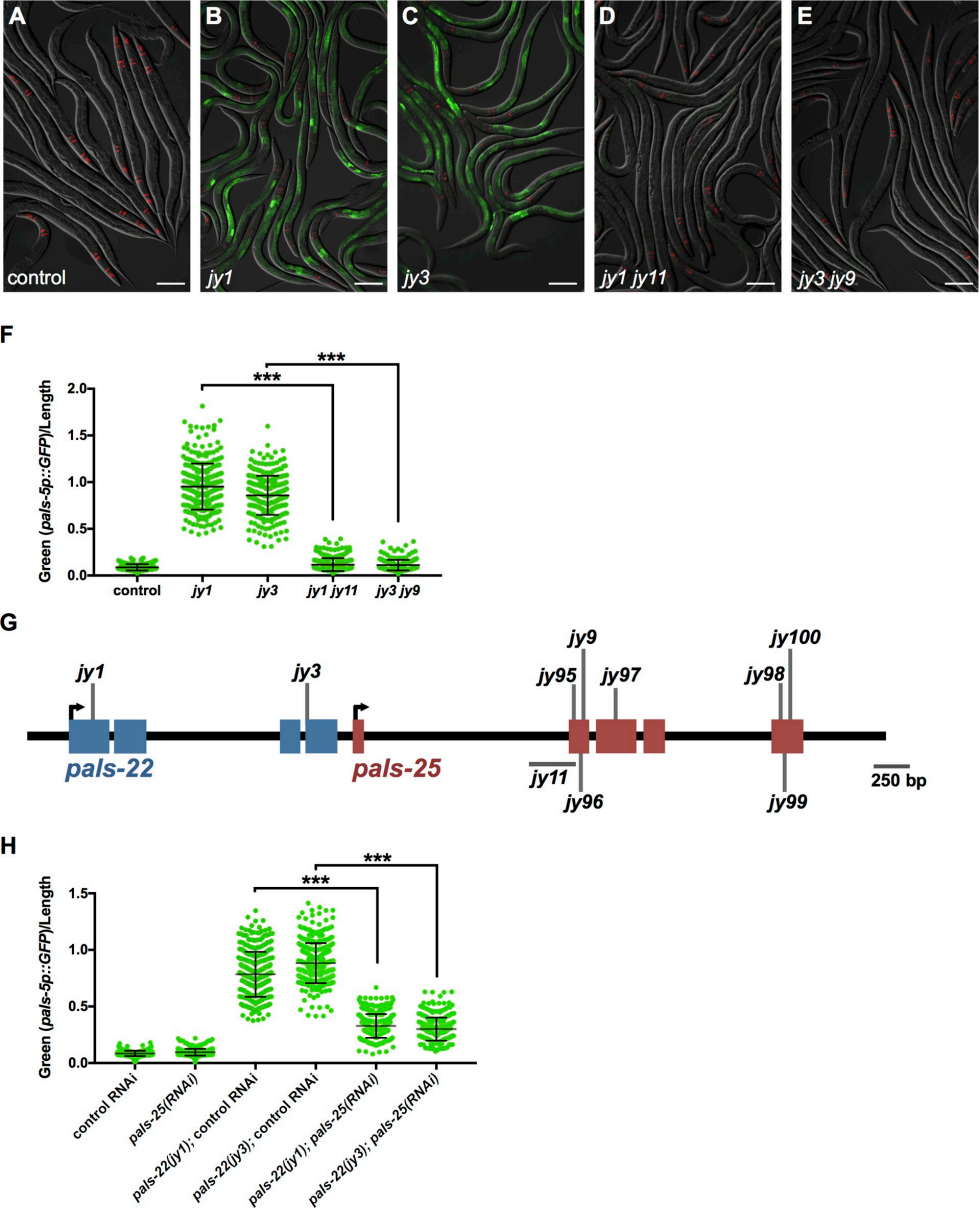
*pals-25* is required for increased *pals-5p*::*GFP* expression in *pals-22* mutants. (A-E) Mutants isolated from *pals-22* suppressor screens show decreased expression of the *pals-5p*::*GFP* reporter. Shown are (A) wild-type, (B) *pals-22(jy1)*, (C) *pals-22(jy3)*, (D) *pals-22(jy1) pals-25(jy11)*, and (E) *pals-22(jy3) pals-25(jy9)* animals. Green is *pals-5p*::*GFP*, red is *myo-2p*::*mCherry* expression in the pharynx as a marker for presence of the transgene. Images are overlays of green, red and Nomarski channels and were taken with the same camera exposure for all. Scale bar, 100 μm. (F) *pals-5p*::*GFP* expression quantified in *pals-22* suppressor mutants using a COPAS Biosort machine to measure the mean GFP signal normalized by length of individual animals, indicated by green dots. Mean signal of the population is indicated by black bars, with error bars as SD. Graph is a compilation of three independent replicates, with at least 100 animals analyzed in each replicate. *** p < 0.001 with Student’s t-test. (G) *pals-22* and *pals-25* gene coding structure (UTRs not shown), with blue exons for *pals-22* and red exons for *pals-25*. See S1 Table “Mutations” sheet for residues altered in the mutated proteins. (H) *pals-5p*::*GFP* expression in animals treated with either L4440 RNAi control or *pals-25* RNAi, quantified using a COPAS Biosort machine to measure the mean GFP signal normalized by length of animals. Parameters the same as in (F).

Previously we found that wild-type *pals-22* represses expression of IPR genes: *pals-22* mutants have constitutive expression of several IPR genes including *pals-5* [6]. A transcriptional reporter consisting of the 1273 bp upstream region of *pals-5* fused to GFP, *pals-5p*::*GFP*, is a reliable marker of IPR gene expression [9] and exhibits strong GFP expression in a *pals-22* mutant background [6] (Fig 1A–1C). To find positive regulators of IPR gene expression, we mutagenized *pals-22; pals-5p*::*GFP* strains and screened for loss of GFP expression in F2 animals. From one screen in the *pals-22(jy1)* mutant background and one screen in the *pals-22(jy3)* mutant background, we screened a total of ~23,000 haploid genomes and found eight independent mutant alleles that almost entirely reverse the increased *pals-5p*::*GFP* expression back to near wild-type levels in *pals-22* mutants (Fig 1A–1F). All of these alleles are recessive, segregate in Mendelian ratios, and fail to complement each other, suggesting they are likely to represent loss-of-function mutations in the same gene.

We used whole-genome sequencing of two mutant strains (*jy9* and *jy100*) to identify the causative alleles [10] and found predicted loss-of-function mutations in *pals-25* in both strains. Further sequencing identified *pals-25* mutations in the remaining six mutant strains (Fig 1G, S1 Table). *pals-25* appears to be in an operon just downstream of *pals-22* (CEOP3012, https://www.wormbase.org), and while these two genes are paralogs, they share limited sequence similarity, with no significant similarity on the DNA level and only 19.4% identity on the amino acid level. Of note, neither *pals-22* nor *pals-25* have obvious orthologs in other *Caenorhabditis* species like *Caenorhabditis briggsae* and *Caenorhabditis brenneri*, and thus appear to be specific to *C*. *elegans* [8]. We used reciprocal BLAST analysis to search for orthologs in the genome of the newly described *C*. *elegans* sister species *Caenorhabditis inopinata* and were unsuccessful (see Materials and Methods). Interestingly, previous analysis of the *pals* gene family indicated that there was no conservation of the *pals-22/25* region with 26 other nematode genomes, with perhaps very weak conservation to the right of *pals-25* [8].

To further confirm that *pals-25* regulates *pals-5p*::*GFP* gene expression in *pals-22* mutants, we performed RNAi against *pals-25* in a *pals-22*; *pals-5p*::*GFP* strain. As expected, we found lowered expression of *pals-5p*::*GFP* (Fig 1H, S1A Fig), indicating that wild-type *pals-25* is required for the increased expression of *pals-5p*::*GFP* seen in a *pals-22* mutant background.

**Fig 2:**
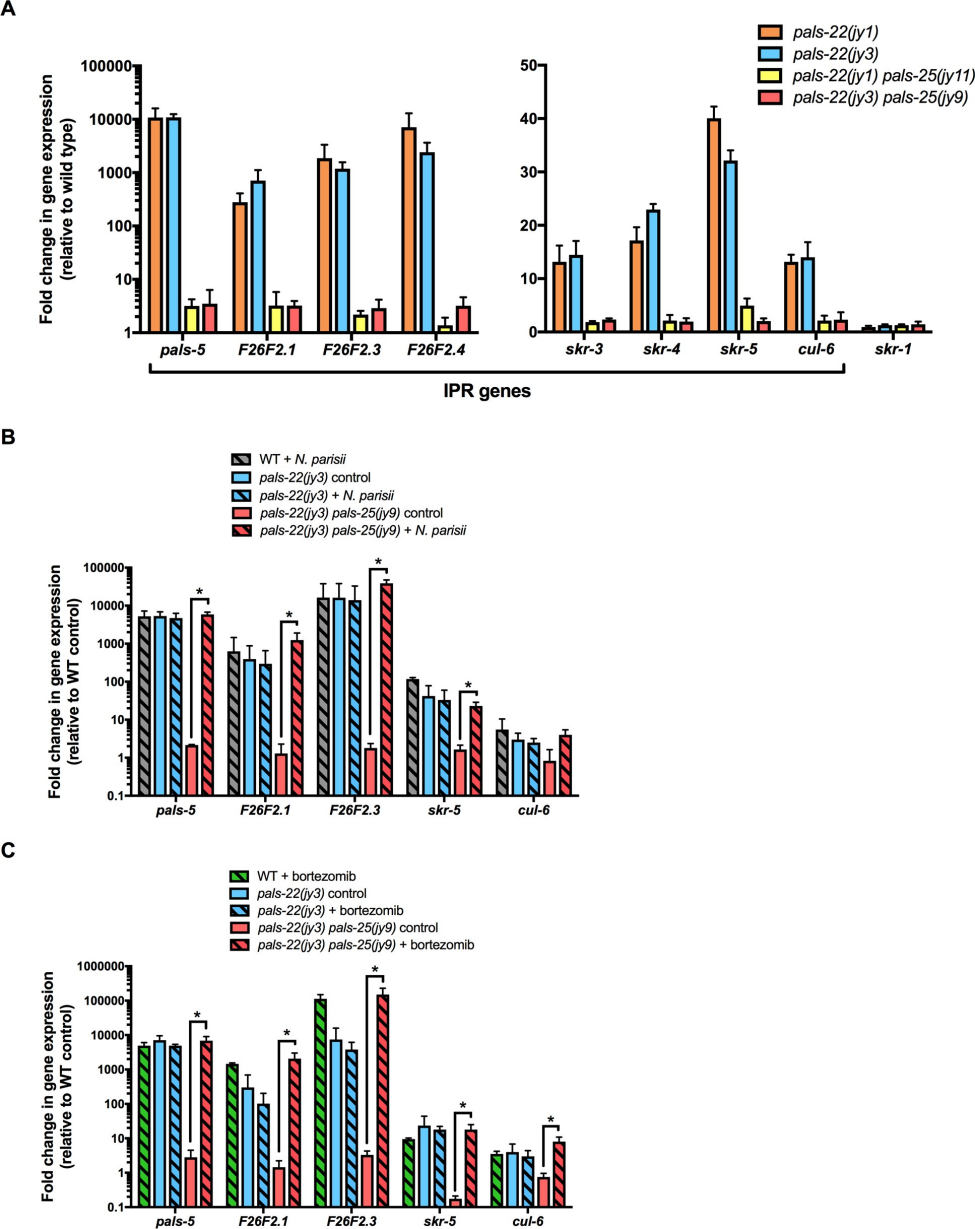
*pals-25* is required for increased IPR gene expression in *pals-22* mutants, but not for IPR induction in response to infection or proteasome inhibition. (A) qRT-PCR measurement of gene expression in *pals-22* and *pals-22 pals-25* animals, shown as the fold change relative to wild-type control. (B-C) qRT-PCR measurement of IPR gene expression in *pals-22(jy3)* and *pals-22(jy3) pals-25(jy9)* animals following 4 hours of infection with *N*. *parisii* (B) or treatment with the proteasome inhibitor bortezomib (C). For (A-C), results shown are the average of two independent biological replicates and error bars are SD. * p < 0.05 with Student’s t-test.

These observations suggest that *pals-25* acts downstream of or in parallel to *pals-22* to activate mRNA expression of IPR genes. We chose two *pals-25* alleles to test this hypothesis in greater detail. Specifically, we used qRT-PCR to measure levels of endogenous mRNA in two different *pals-22 pals-25* mutants compared to two different *pals-22* mutants and wild-type animals (Fig 2A). We analyzed mRNA levels of *pals-5*, as well as seven other IPR genes including nematode-specific genes of unknown function (F26F2.1, F26F2.3, and F26F2.4) and predicted ubiquitin ligase components *skr-3*, *skr-4*, *skr-5* and *cul-6*. Here we found that mutations in *pals-25* reverse the elevated mRNA levels of all eight of these IPR genes in a *pals-22* mutant background back to near wild-type levels. Importantly, a non-IPR gene, *skr-1*, is not affected by mutations in *pals-22* or *pals-25*. These results indicate that in a *pals-22* mutant background, wild-type *pals-25* is required to activate IPR gene expression.

Previous analysis indicated that *pals-22* is broadly expressed in several tissues in the animal, including the intestine and the epidermis [6, 8]. Similarly, we found that *pals-25* is broadly expressed. Using a transgenic strain with a fosmid containing *pals-25* with endogenous *cis* regulatory control and tagged at the C terminus with GFP and 3xFLAG [11], we observed PALS-25::GFP expression throughout the animal, including expression in the neurons, epidermis, and intestine (S1B Fig). We did not see any change in PALS-25::GFP expression after *pals-22* RNAi treatment (S1C Fig), suggesting that PALS-22 is unlikely to regulate mRNA or protein levels of PALS-25.

### IPR genes are induced by infection and by proteasome blockade in *pals-22 pals-25* mutants

As *pals-25* is required to activate IPR gene expression in a *pals-22* mutant background, we wondered whether *pals-25* was also required for inducing IPR gene expression in response to external triggers. We originally identified IPR genes because of their induction by *N*. *parisii* infection [6, 9], which is an intracellular pathogen in the Microsporidia phylum that invades and undergoes its entire replicative life cycle inside *C*. *elegans* intestinal cells [12]. We therefore infected animals with *N*. *parisii* and compared induction of IPR genes in *pals-22 pals-25* mutants and wild-type animals at 4 hours (Fig 2B). Here we found similar levels of IPR gene induction in *pals-22 pals-25* and wild-type animals, suggesting that *pals-22*/*25* regulate expression of IPR genes independently of infection. Next, we examined the role of *pals-22*/*25* in the transcriptional response to proteasome blockade, which is another trigger of IPR gene expression [9] (Fig 2C). We used bortezomib, which is a small molecule inhibitor of the 26S proteasome. Here again, we found that bortezomib treatment induced IPR gene expression in *pals-22 pals-25* mutants at levels similar to wild-type animals. Therefore, *pals-22/25* appear to regulate IPR gene expression independently of infection and proteasomal stress.

### *pals-25* mutations reverse multiple physiological phenotypes caused by *pals-22* mutations

**Fig 3:**
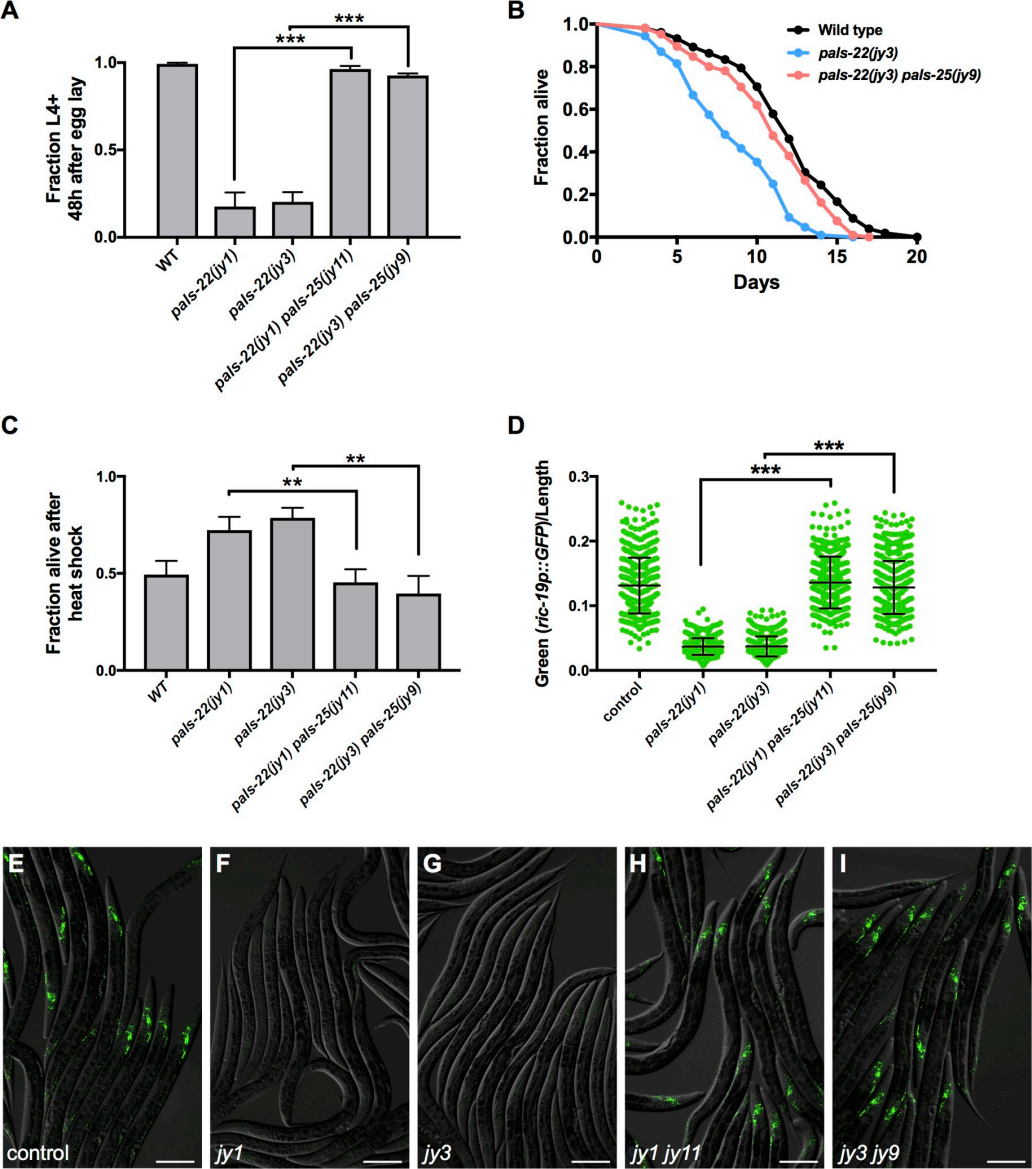
*pals-25* mutations suppress diverse phenotypes of *pals-22* mutants. (A) *pals-25* mutation suppresses the developmental delay of *pals-22* mutants. Fraction of animals reaching the L4 larval stage 48 hours after eggs were laid is indicated. Results shown are the average of three independent biological replicates, with 100 animals assayed in each replicate. Error bars are SD. *** p < 0.001 with Student’s t-test. (B) Lifespan of wild type, *pals-22(jy3)*, and *pals-22(jy3) pals-25(jy9)* animals. Assays were performed with 40 animals per plate, and three plates per strain per experiment. This experiment was repeated three independent times with similar results, with data from a representative experiment shown. See S2A and S2B Fig for other replicates. p-value for *pals-22(jy3)* compared to *pals-22(jy3) pals-25(jy9)* is <0.0001 using the Log-rank test. (C) The increased survival of *pals-22* mutants after heat shock is suppressed by loss of function mutations in *pals-25*. Animals were incubated for 2 hours at 37°C followed by 24 hours at 20°C, and then assessed for survival. Strains were tested in triplicate, with at least 30 animals per plate. Mean fraction alive indicates the average survival among the triplicates, errors bars are SD. ** p < 0.01. Assay was repeated three independent times with similar results, and data from a representative experiment are shown. See S2C and S2D Fig for other replicates. (D-I) *pals-25* mutation suppresses transgene silencing in *pals-22* mutants. (D) *ric-19p*::*GFP* expression quantified in *pals-22* and *pals-22 pals-25* mutants using a COPAS Biosort machine to measure the mean GFP signal and length of individual animals, indicated by green dots. Mean signal of the population is indicated by black bars with error bars as SD. Graph is a compilation of three independent replicates, with at least 100 animals analyzed in each replicate. *** p < 0.001 with Student’s t-test. In (E-I), green is neuronal expression of *ric-19p*::*GFP*. Shown are (E) wild-type, (F) *pals-22(jy1)*, (G) *pals-22(jy3)*, (H) *pals-22(jy1) pals-25(jy11)*, and (I) *pals-22(jy3) pals-25(jy9)* animals. Images are overlays of green and Nomarski channels and were taken with the same camera exposure for all. Scale bar, 100 μm.

*pals-22* mutants have several striking physiological phenotypes, including slowed growth and shorter lifespans, as well as increased resistance to proteotoxic stress like heat shock [6]. Therefore, we investigated whether mutations in *pals-25* suppress these phenotypes of *pals-22* mutants. First, we investigated developmental rate by measuring the fraction of animals that reach the fourth larval (L4) stage by 48 hours after embryogenesis. Nearly all wild-type animals are L4 at this timepoint, whereas less than 20% of *pals-22* mutants are L4 (Fig 3A). We found that mutations in *pals-25* completely reverse this delayed development of *pals-22* mutants, with nearly all *pals-22 pals-25* mutants reaching the L4 stage by 48 hours (Fig 3A). Next, we analyzed lifespan, as previous work showed that *pals-22* mutants have a significantly shortened lifespan compared to wild-type animals [6, 8]. Here again we found that *pals-25* mutations reversed this effect, with *pals-22 pals-25* mutants having lifespans comparable to wild-type animals (Fig 3B, S2A and S2B Fig). Next, we investigated the effect of *pals-25* mutations on the thermotolerance capacity of *pals-22* mutants, which is greatly enhanced compared to wild-type animals. We found that *pals-22 pals-25* double mutants have survival after heat shock at levels similar to wild-type animals (Fig 3C, S2C and S2D Fig), indicating that *pals-25* is required for the enhanced thermotolerance of *pals-22* mutants. Thus, these results show that in a *pals-22* mutant background, wild-type *pals-25* is required to delay development, shorten lifespan and enhance thermotolerance.

Our previous results suggested that *pals-22* acts independently of the canonical heat shock response controlled by the transcription factor HSF-1 [6, 9], which also regulates thermotolerance as well as pathogen resistance [13–15]. Interestingly, we found that a *hsf-1(sy441); pals-22(jy1)* double mutant has a larval arrest phenotype, suggesting that *hsf-1* and *pals-22* regulate parallel pathways that converge on some essential process [6]. To determine whether *pals-25* acts downstream of *pals-22* for this phenotype we constructed an *hsf-1(sy441); pals-22(jy1) pals-25(jy11)* triple mutant and found that this strain is viable. Therefore, *pals-25* acts downstream of *pals-22* to suppress synthetic lethality with *hsf-1(sy441)*.

Previous work from the Hobert lab identified *pals-22* in a screen for regulators of reporter gene expression in neurons [8]. They found that mutations in *pals-22* led to decreased levels of GFP reporter expression in neurons and other tissues, and wild-type *pals-22* thus acts as an ‘anti-silencing’ factor of multi-copy transgene expression. Therefore, we analyzed the effects of *pals-25* mutations on transgene silencing in *pals-22* mutants. Here we found that *pals-25* mutations reverse the enhanced silencing of a neuronally expressed GFP transgene in *pals-22* mutants (Fig 3D–3I), indicating that wild-type *pals-25* activity is required to silence expression from multi-copy transgenes in a *pals-22* mutant background. Of note, previous work found that a *pals-25* mutation alone does not affect transgene silencing [8]. In summary, loss of function mutations in *pals-25* appear to fully reverse all previously described phenotypes of *pals-22* mutants.

### *pals-22* mutants have immunity against coevolved intestinal pathogens of the intestine, which is suppressed by *pals-25* mutations

**Fig 4:**
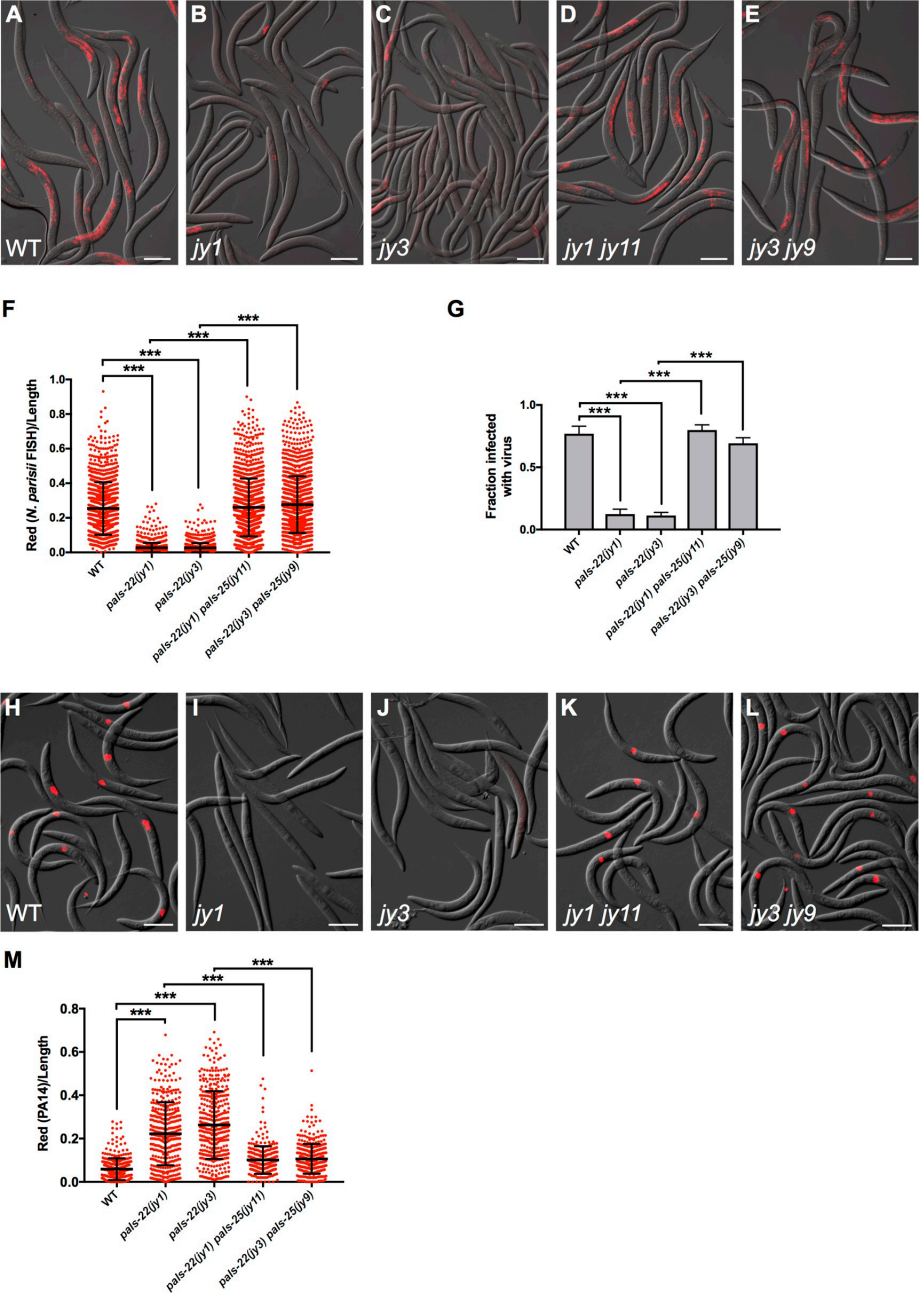
*pals-22* mutants have increased resistance to infection by *N*. *parisii* or Orsay virus, dependent on *pals-25*. (A-E) Images of (A) wild-type, (B) *pals-22(jy1)*, (C) *pals-22(jy3)*, (D) *pals-22(jy1) pals-25(jy11)*, and (E) *pals-22(jy3) pals-25(jy9)* animals infected with *N*. *parisii* as L1s, fixed 30 hours post infection, and stained by FISH with an *N*. *parisii*-specific probe (red). Scale bar, 100 μm. (F) *N*. *parisii* FISH signal quantified using a COPAS Biosort machine to measure the mean red signal normalized to the length of individual animals, indicated by red dots. Mean signal of the population is indicated by black bars, with error bars as SD. Graph is a compilation of three independent replicates, with at least 100 animals analyzed in each replicate. *** p < 0.001 with Student’s t-test. (G) Fraction of animals infected with the Orsay virus 18 hours post infection is indicated. Animals were fixed and stained by FISH with a virus-specific probe, and scored visually for infection. Results shown are the average of three independent biological replicates, with 100 animals assayed in each replicate. Error bars are SD. *** p < 0.001 with Student’s t-test. (H-L) Images of (H) wild-type, (I) *pals-22(jy1)*, (J) *pals-22(jy3)*, (K) *pals-22(jy1) pals-25(jy11)*, and (L) *pals-22(jy3) pals-25(jy9)* animals infected with the Orsay virus as L1s, fixed 18 hours post infection, and stained by FISH with a virus-specific probe (red). Scale bar, 100 μm. (M) Quantification of dsRed fluorescence levels in wild-type, *pals-22*, and *pals-22 pals-25* animals after 16 hours of exposure to dsRed-expressing PA14. Red fluorescence was measured using a COPAS Biosort machine to measure the mean red signal and length of individual animals, indicated by red dots. Mean signal of the population is indicated by black bars, with error bars as SD. Graph is a compilation of three replicates, with at least 100 animals analyzed in each replicate. *** p < 0.001 with Student’s t-test.

In addition to the previously described phenotypes of *pals-22* mentioned above, we analyzed resistance of these mutants to intracellular infection. First, we analyzed the resistance of *pals-22* mutants to *N*. *parisii* infection. We fed animals a defined dose of microsporidia spores and measured pathogen load inside intestinal cells. We analyzed pathogen load at 30 hours post infection (hpi), when *N*. *parisii* is growing intracellularly in the replicative meront stage, and found greatly lowered pathogen load in *pals-22* mutants compared to wild-type animals (Fig 4A–4F). We then tested *pals-22 pals-25* double mutants and found these animals to have resistance comparable to wild type. One explanation for the altered levels of *N*. *parisii* observed in the intestines of *pals-22* mutant animals is that these mutants have lowered feeding or accumulation of pathogen in the intestine, and thus simply have a lower exposure to *N*. *parisii*. To address this concern, we added fluorescent beads to our *N*. *parisii* infection assay and measured accumulation in the intestinal lumen. Here we found that *pals-22* mutants and *pals-22 pals-25* double mutants accumulated fluorescent beads at comparable levels to wild-type animals (S3A Fig), suggesting that their pathogen resistance to *N*. *parisii* is not simply due to lowered exposure to the pathogen in the intestinal lumen. As a positive control in this assay we tested *eat-2(ad465)* mutants and found that they had reduced fluorescent bead accumulation, consistent with their previously characterized feeding defect [16]. Altogether, these results indicate that *pals-22* and *pals-25* regulate resistance to infection by microsporidia.

We also investigated resistance of *pals-22* mutants and *pals-22 pals-25* double mutants to other pathogens. First, we measured resistance to infection by the Orsay virus. Like *N*. *parisii*, Orsay virus is a natural pathogen of *C*. *elegans* and replicates inside of *C*. *elegans* intestinal cells [17, 18]. We used FISH staining of Orsay viral RNA to quantify the fraction of worms infected at 18 hpi. Here we found that *pals-22* mutants had significantly decreased viral load when compared to wild-type animals (Fig 4G–4L). This lowered viral infection in *pals-22* mutants was fully reversed to wild-type levels in *pals-22 pals-25* mutants. Importantly, we confirmed that *pals-22* and *pals-22 pals-25* mutants do not have altered fluorescent bead accumulation in the intestine compared to wild-type animals in the presence of virus (S3B Fig), indicating that their lowered viral load is not likely due to lowered exposure to the virus.

Interestingly, we found that *pals-22* mutants did not have reduced pathogen load when infected with the Gram-negative bacterial pathogen *Pseudomonas aeruginosa* (clinical isolate PA14) (Fig 4M). In fact, these mutants had increased pathogen load, which was suppressed by mutations in *pals-25*. To our knowledge, *P*. *aeruginosa* species are not common pathogens of nematodes in the wild, although under laboratory conditions, *P*. *aeruginosa* PA14 does accumulate in the *C*. *elegans* intestinal lumen and causes a lethal infection [19, 20]. In summary *pals-22* mutants have increased resistance to natural pathogens of the intestine but increased susceptibility to PA14, a ‘non-natural’ pathogen of the intestine.

**Fig 5:**
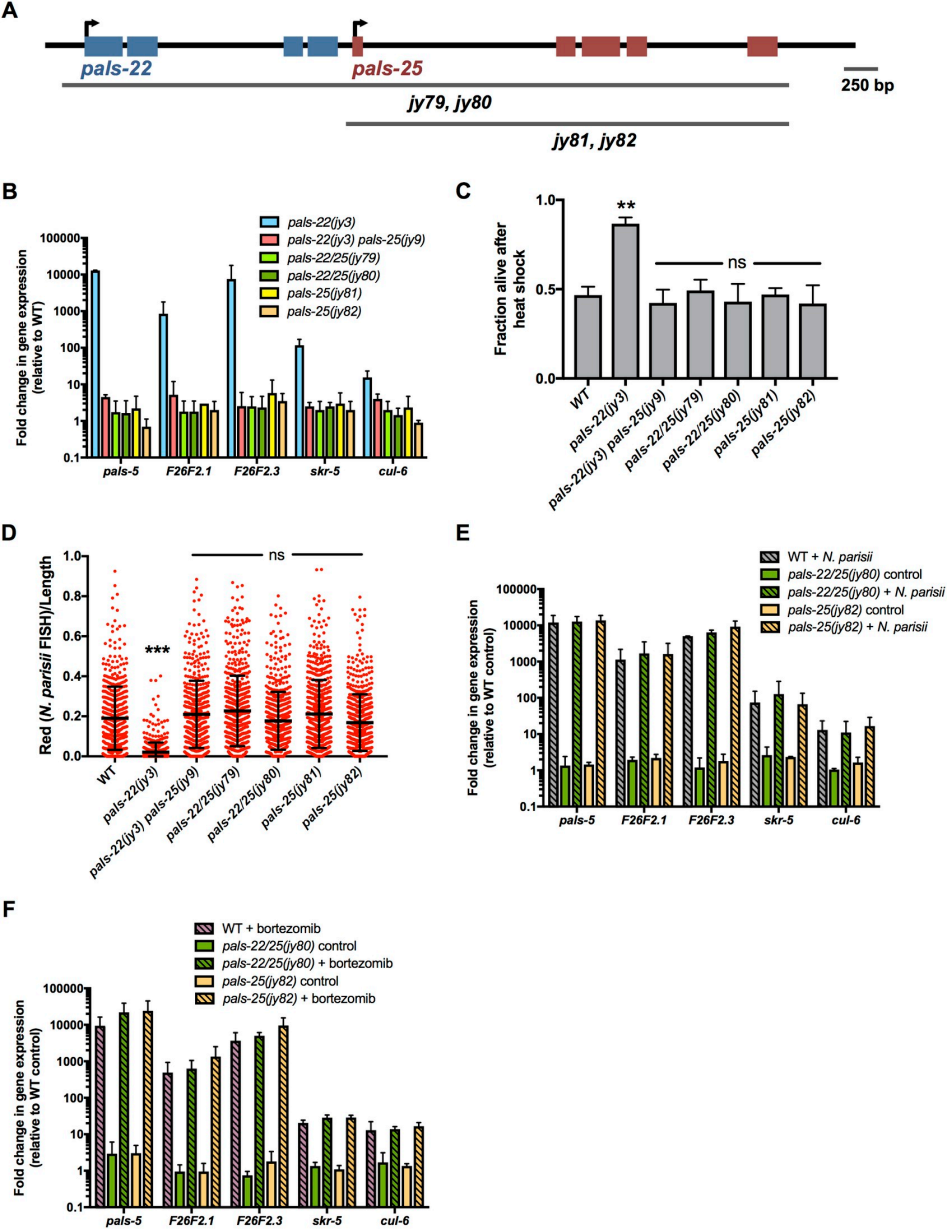
A complete deletion of the *pals-25* gene and of the *pals-22/25* locus leads to wild-type phenotypes. (A) *pals-22* and *pals-25* gene coding structure (UTRs not shown), with blue exons for *pals-22* and red exons for *pals-25*. Deleted regions are indicated; see S1 Table for more information. (B) qRT-PCR measurement of gene expression, shown as the fold change relative to wild-type control. (C) Survival of animals after 2 hour heat shock treatment at 37°C followed by 24 hours at 20°C. Strains were tested in triplicate, with at least 30 animals per plate. Assay was repeated three independent times with similar results, and data from a representative experiment are shown. Mean fraction alive indicates the average survival among the triplicates, errors bars are SD. ** p < 0.01, ns, not significant with Student’s t-test as compared to wild-type control. (D) *N*. *parisii* FISH signal quantified using a COPAS Biosort machine to measure the mean red signal normalized by length of individual animals, indicated by red dots. Mean signal of the population is indicated by black bars, with error bars as SD. Graph is a compilation of three independent replicates, with at least 100 animals analyzed in each replicate. *** p < 0.001, ns, not significant with Student’s t-test as compared to wild-type control. (E-F) qRT-PCR measurement of IPR gene expression in *pals-22/25(jy80)* and *pals-25(jy82)* animals following 4 hours of infection with *N*. *parisii* (E) or treatment with the proteasome inhibitor bortezomib (F). For (B,E-F), results shown are the average of two independent biological replicates and error bars are SD.

The results above indicate that *pals-25* acts downstream or in parallel to *pals-22* to regulate IPR phenotypes (Figs 1–4). If *pals-25* were acting downstream of *pals-22*, the prediction is that a *pals-25* mutation in a wild-type background would have no effect. Because of the close genomic proximity of *pals-22* and *pals-25*, it would be extremely difficult to recombine our existing *pals-25* mutations away from *pals-22* mutations into a wild-type background. Therefore, we used CRISPR/Cas9 editing to generate two independent *pals-25* deletions in otherwise wild-type backgrounds (Fig 5A). In both cases we found that these *pals-25* null mutants have wild-type levels of IPR gene expression, thermotolerance and *N*. *parisii* pathogen load (Fig 5B–5D). In addition, we used CRISPR/Cas9 editing to delete the entire *pals-22 pals-25* locus and analyzed two independent alleles. In both cases we found that these *pals-22/25* null mutants have wild-type levels of IPR gene expression, thermotolerance and *N*. *parisii* pathogen load (Fig 5B–5D), consistent with our previous analyses of *pals-22 pals-25* mutants isolated from forward genetic screens. In addition, IPR gene expression was still induced by *N*. *parisii* or bortezomib treatment in these mutants (Fig 5E and 5F). These results are all consistent with *pals-25* acting downstream of *pals-22* and indicate that the entire *pals-22/25* locus is dispensable for IPR induction, thermotolerance, and resistance to *N*. *parisii*.

### RNA-seq analysis of *pals-22/25*-upregulated genes define the IPR

**Fig 6:**
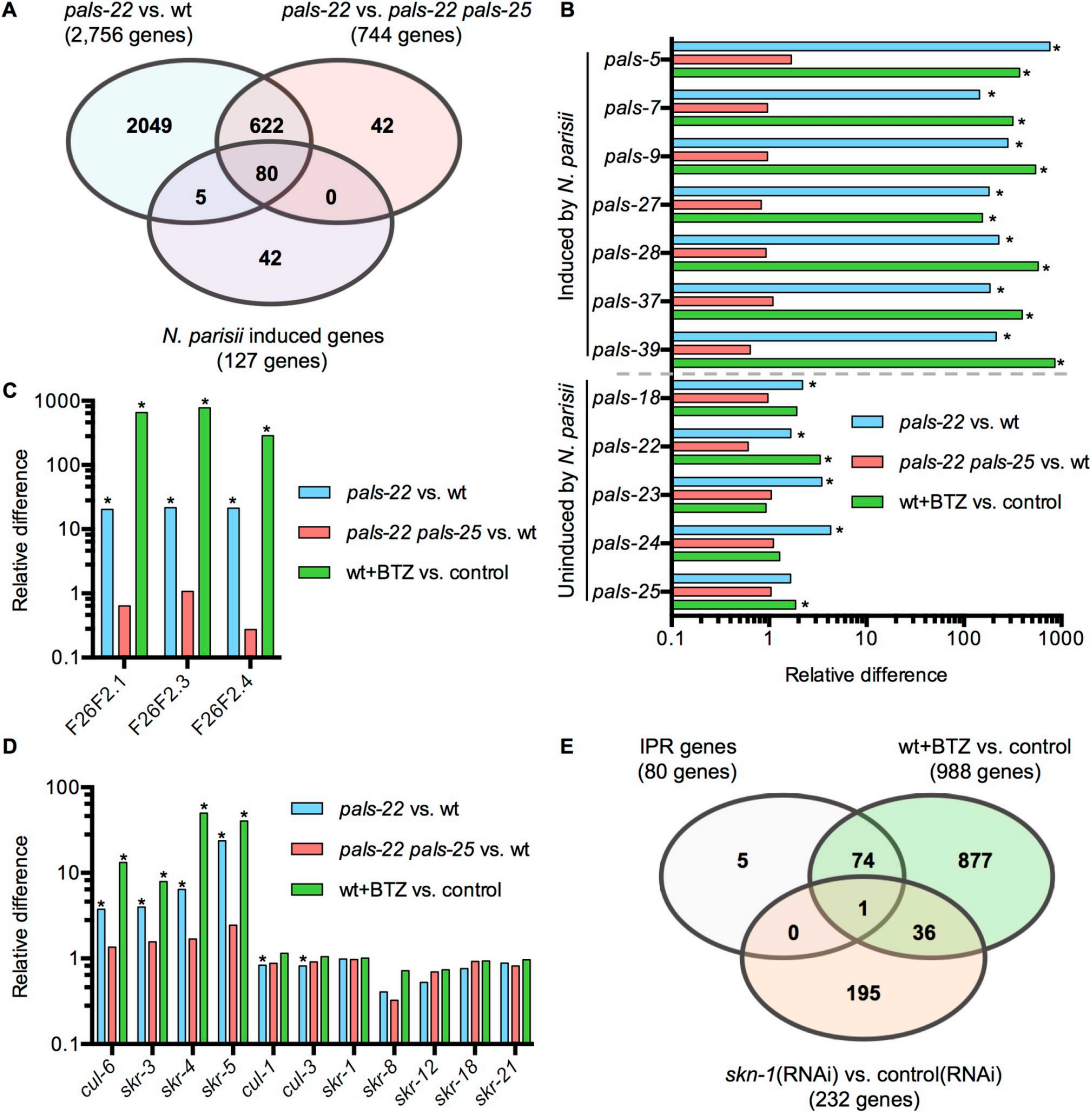
The *pals-22/25* gene pair transcriptionally regulates genes that are induced by *N*. *parisii* infection or proteasome blockade. (A) Venn diagram comparing 1) genes upregulated in *pals-22* mutants compared to wild-type animals, 2) genes upregulated in *pals-22* mutants compared to *pals-22 pals-25* double mutants and 3) genes induced in wild-type animals in response to *N*. *parisii* infection. Gene sets 1 and 2 were obtained from RNA-seq data outlined in this study, and Gene set 3 was obtained via RNA-seq in a previous study [9]. Gene sets 1 and 2 have a highly significant overlap (rf = 3.9; p<1.0E-317), gene sets 1 and 3 significantly overlap (rf = 2.8; p<1.41E-24), as do gene sets 2 and 3 (rf = 9.6; p<2.48E-63). We define the IPR genes as the 80 genes common across the three gene sets. (B-D) The relative mRNA levels measured by RNA-seq in: *pals-22(jy3)* animals compared to wild-type; *pals-22(jy3) pals-25(jy9)* animals compared to wild-type; and wild-type animals treated with the proteasome inhibitor bortezomib (BTZ) compared to DMSO (vehicle control for BTZ). * FDR<0.01 as calculated by edgeR and limma (see Materials and Methods) indicates the gene is considered to be differentially expressed. (B) *pals* genes induced by *N*. *parisii* infection are also regulated by *pals-22/25* and induced by BTZ. (C) Species-specific F26F2 genes are regulated by the *pals-22/25* gene pair and are induced by BTZ. (D) The Cullin-Ring Ligase components Cullin (*cul)* and Skp-related (*skr)* genes that are upregulated during *N*. *parisii* infection are also regulated by the *pals-22/25* gene pair and induced by BTZ. (E) Venn Diagram showing overlap between: IPR genes defined in (A); genes upregulated due to treatment with BTZ; and genes upregulated by *skn-1*[21]. The overlap between IPR genes and genes induced following BTZ treatment is highly significant (rf = 10.8; p<2.68E-74), while the overlap between IPR genes and SKN-1 regulated genes is that expected by random chance (rf = 0.6; p<0.487). The overlap between BTZ-induced genes and SKN-1 regulated genes is considered significant (rf = 1.8; p<2.04E-4). See S2 and S3 Tables for detailed expression levels of genes discussed here.

Previous work indicated that *N*. *parisii* and the Orsay virus induce a common set of genes, despite these being very different pathogens [9]. We called eight of these genes the IPR subset [6], and here we show they are regulated by *pals-22/25* (Fig 2A). To identify additional genes regulated by *pals-22/25*, we performed RNA-seq analysis of *pals-22* mutants, *pals-22 pals-25* mutants, and wild-type animals. We performed differential gene expression analysis using a FDR<0.01 cutoff (see Materials and Methods for a complete description of criteria) and determined that 2,756 genes were upregulated in *pals-22* mutants compared to wild-type animals (Fig 6A, S4 Table). Next, we compared *pals-22* mutants to *pals-22 pals-25* double mutants and found that 744 genes were upregulated (Fig 6A, S4 Table). Of these two comparisons, there are 702 genes in common that are upregulated both in *pals-22* mutants compared to wild-type animals and in *pals-22* mutants compared to *pals-22 pals-25* double mutants (Fig 6A). Therefore, these 702 genes are negatively regulated by wild-type *pals-22* and require the activity of the wild-type *pals-25* for their induction in the absence of wild-type *pals-22*. These 702 genes include *pals-5* (Fig 1) and other IPR genes (Fig 2).

Because *pals-22* is upstream of *pals-25* in an operon, we investigated whether a *pals-22* mutation might affect mRNA expression of *pals-25*. Consistent with the lack of PALS-25::GFP changes upon *pals-22* RNAi treatment, we found no significant difference between *pals-25* mRNA expression in *pals-22(jy3)* mutants compared to wild-type animals (Fig 6B). To investigate whether a *pals-25* mutation had an effect on *pals-22* mRNA expression we performed qRT-PCR on a *pals-25(jy81)* deletion mutant and here we found there was no significant effect on *pals-22* mRNA expression in these mutants compared to wild-type animals (S4 Fig).

We next compared the 702 *pals-22/25*-regulated genes to genes induced during *N*. *parisii* infection identified in a previous study [9] to expand our list of IPR genes. Out of 127 genes induced during *N*. *parisii* infection, we found that the *pals-22/25* gene pair regulated mRNA expression of 80 of these genes (Fig 6A). Specifically, of the 25 *pals* genes induced upon intracellular infection, all are induced in *pals-22* mutants and reverted back to wild-type levels in *pals-22 pals-25* double mutants (Fig 6B, S4 Table). Notably, all *pals* genes that are not regulated by *pals-22/25* are also not induced by infection. Furthermore, the other nematode-specific genes F26F2.1, F26F2.3, and F26F2.4, which are induced by *N*. *parisii* and Orsay virus infection, were also found to be induced in *pals-22* mutants and brought back to wild-type levels in *pals-22 pals-25* double mutants (Fig 6C). In addition, we found that the ubiquitin ligase components are similarly regulated (Fig 6D). These studies thus define IPR genes as the 80 genes that are: 1) induced by *N*. *parisii* infection, 2) induced in a *pals-22* mutant background, and 3) reversed back to wild-type levels in *pals-22 pals-25* double mutants.

### IPR genes are also regulated by proteasomal stress

Previous work indicated that blockade of the proteasome, either pharmacologically or genetically, will induce expression of a subset of IPR genes [9]. To determine the IPR genes that are induced by proteasome stress, we performed RNA-seq analysis to define the whole-genome response to this stress. Again, we conducted differential expression analysis and compared gene expression of animals after 4 hours of exposure to the proteasome inhibitor bortezomib compared to the DMSO vehicle control. From these experiments we determined that 988 genes are induced following bortezomib treatment, using the cut-off mentioned above and described in the Materials and Methods. Interestingly, nearly all of the IPR genes described above are also induced following bortezomib treatment (Fig 6E), an overlap that is highly significant (representation factor (rf) = 10.8; p<2.68E-74; rf is the ratio of actual overlap to expected overlap where rf>1 indicates overrepresentation and rf<1 indicates underrepresentation; see Materials and Methods). Previous work has shown that genes induced by *N*. *parisii* do not include the proteasome subunits induced by proteasome blockade as part of the bounceback response [9]. The bounceback response is induced via the transcription factor SKN-1. Consistent with these results, here we find that the IPR genes induced by bortezomib are distinct from those regulated by the transcription factor SKN-1, as defined by a previous study [21]. The overlap between SKN-1 regulated genes and IPR genes includes only one gene (Fig 6E), a number expected by random chance (rf = 0.6; p<0.487).

As shown earlier, *pals-22* mutants have increased resistance to heat shock, and previous work indicated that there is overlap between genes induced by chronic heat stress and genes induced by *N*. *parisii* and virus infection [9]. However, the genes in common are distinct from the canonical chaperones, or Heat Shock Proteins (HSPs), which are induced by the heat shock transcription factor HSF-1. To learn more about the connection between heat shock response, HSF-1, and the IPR, we compared the IPR genes with those induced by HSF-1 as defined in a previous study [22]. Here we found no genes in common between our set of 80 IPR genes and the set of 365 genes upregulated by HSF-1 (rf = 0; p<0.073) (S5 Table). We also compared the 365 genes upregulated by HSF-1 with the 702 genes that are regulated by *pals-22/25* and found 16 genes in common (rf = 0.7; p<0.088) (S5 Table). These are approximately the numbers expected by random chance and indicate that *pals-22/25* regulate a distinct set of genes compared to those regulated by HSF-1.

### *pals-22* and *pals-25* regulate expression of genes induced by other natural pathogens

**Fig 7:**
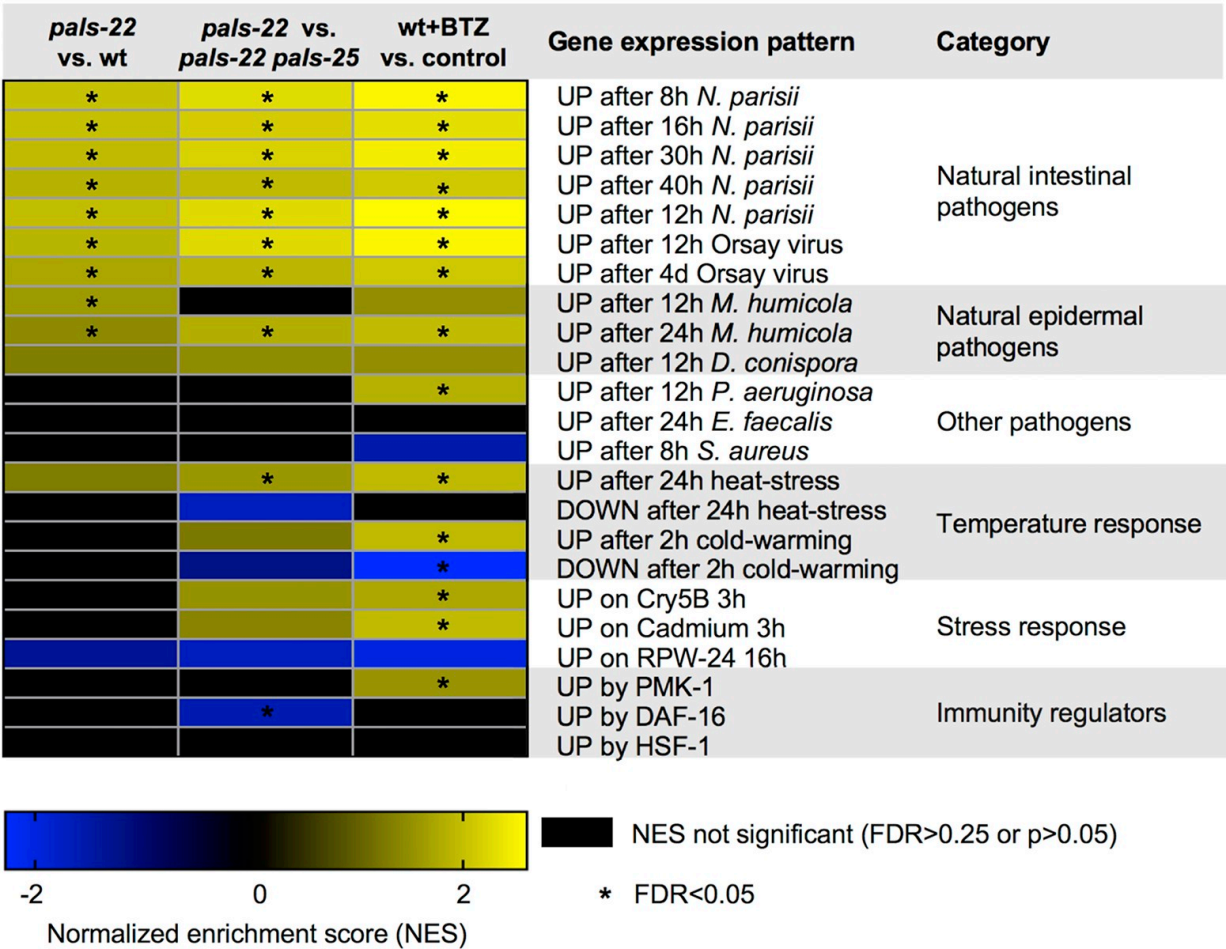
Functional enrichment analysis of genes transcriptionally regulated by the *pals-22/25* gene pair. Correlation of genes transcriptionally regulated by the *pals-22/25* gene pair and genes differentially expressed due to bortezomib treatment with genes that are up- or downregulated in response to infection by pathogens or other environmental stresses. Analysis was performed using the GSEA 3.0 software package (see Materials and Methods) and correlation is quantified as a Normalized Enrichment Score (NES). A positive NES (yellow) indicates correlation with upregulated genes in the denoted comparison while a negative NES (blue) indicates correlation with downregulated genes. Black cells indicate no significant correlation was detected, a FDR>0.25, or p>0.05). *FDR<0.05. For more detailed results, see S7 Table. For details on the gene sets used see S6 Table.

Next, we used Gene Set Enrichment Analysis (GSEA) to broadly compare *pals-22/25*-regulated genes to genes regulated by other pathogens, stressors, and stress-related pathways. Here we found that *pals-22/25* does not significantly regulate expression of genes induced by the Gram-negative bacterial pathogen *P*. *aeruginosa* or the Gram-positive bacterial pathogens *Staphylococcus aureus* and *Enterococcus faecalis* as analyzed in previous studies (Fig 7). Notably, the strains used in these studies are clinical isolates. Furthermore, these pathogen species are not known to be natural pathogens of nematodes and are not found inside *C*. *elegans* intestinal cells before there is extensive tissue damage in the host [23]. (Refer to S7 Table for additional comparisons among genes regulated by *pals-22/25*, bortezomib treatment, and other pathogens and stress pathways.)

Because *pals-22* and *pals-25* regulate expression of a majority of the genes induced by natural intestinal pathogens like *N*. *parisii* and the Orsay virus, we investigated whether they regulate the transcriptional response to natural pathogens that infect other tissues. The fungal pathogen *Drechmeria coniospora* infects and penetrates the epidermis of nematodes, triggering a GPCR signaling pathway that upregulates expression of neuropeptide-like (*nlp*) genes including *nlp-29* to promote defense [19]. Our transcriptome analysis shows that *pals-22/25* regulates a number of genes in common with *Drechmeria* infection (rf = 0.8; p<0.009), though this number is approximately what is expected by chance (S5 Table). Notably, these genes do not include the well-characterized neuropeptide *nlp* defense genes, although they do include many of the *pals* genes. A more recently described natural pathogen of *C*. *elegans* is *Myzocytiopsis humicola*, which is an oomycete that also infects through the epidermis and causes a lethal infection [24]. Here, *pals-22/25* regulate a significant number of genes in common (rf = 4.4; p<5.68E-11) with those induced by *M*. *humicola* 24h post infection, including the chitinase-like ‘*chil’* genes that promote defense against this pathogen (S5 Table). Interestingly, these *chil* genes, like the *pals* genes, are species-specific [8, 24].

We next used OrthoList 2 [25] to determine which genes identified from our RNA-seq analyses have predicted human orthologs. Of the 702 genes regulated by *pals-22/25*, 279 genes (39.7%) have predicted human orthologs (S8 Table), which is somewhat fewer genes than expected by chance (rf = 0.7; p<9.15e-20). In contrast, of the 368 genes induced in *hsf-1* mutants, 190 (51.6%) have predicted human orthologs, which is approximately what is expected by chance (rf = 0.9; p<0.04). Therefore, more of the genes regulated by the conserved transcription factor *hsf-1* have human orthologs compared to genes regulated by the *C*. *elegans*-specific *pals-22/25* gene pair. Furthermore, when we restrict our analysis to just the 80 IPR genes, only 14 (17.5%) have predicted human orthologs, which is far fewer than expected by chance (rf = 0.3; p<9.82e-13), indicating that the transcriptional response to natural infection is significantly enriched for genes that are not well-conserved.

### *pals-22/25* control expression of epidermal defense genes induced by oomycetes

**Fig 8:**
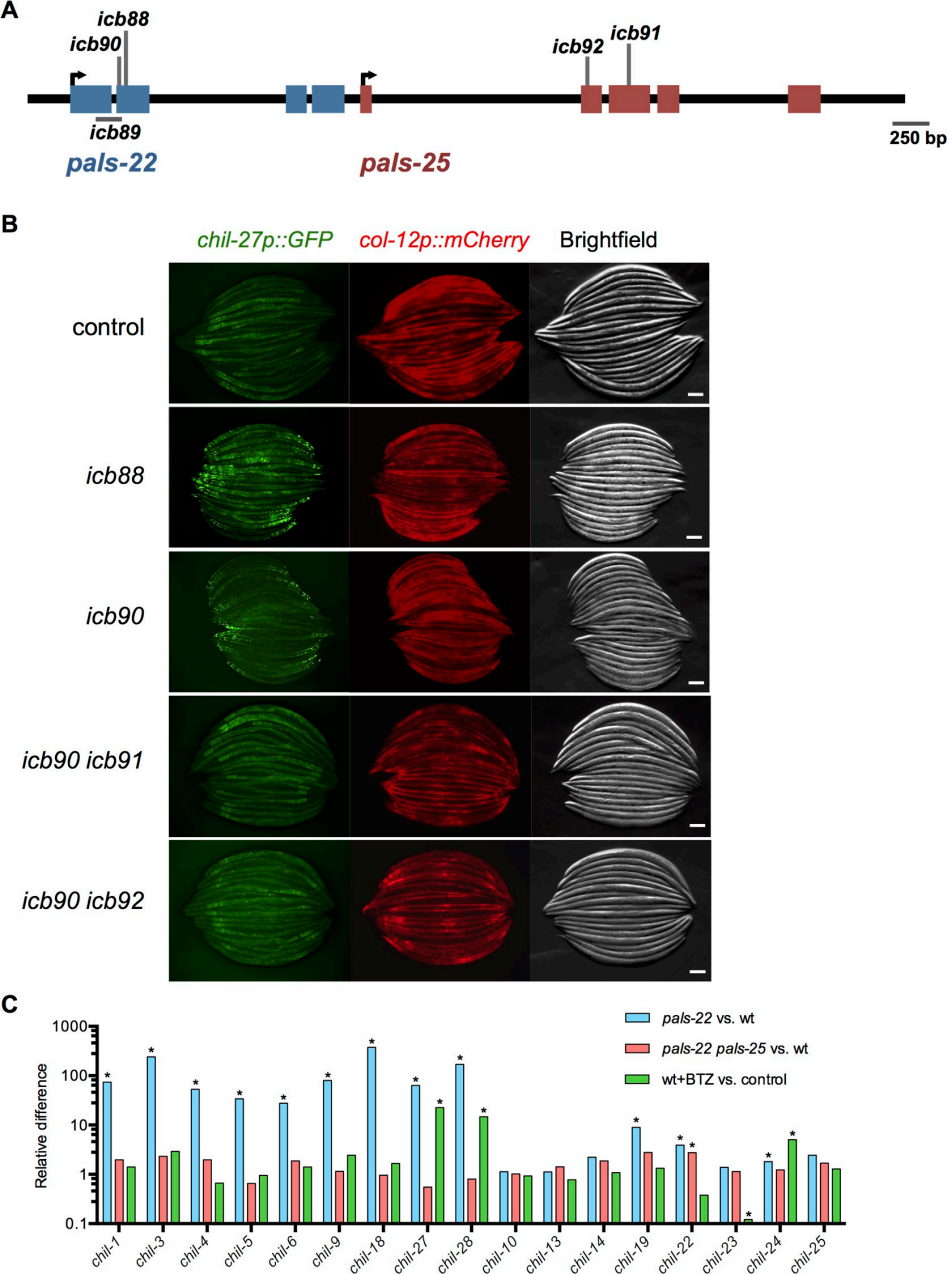
*pals-22* and *pals-25* regulate expression of *chil-27* in the epidermis. (A) *pals-22* and *pals-25* gene coding structure (UTRs not shown), with blue exons for *pals-22* and red exons for *pals-25*. See S1 Table for residues altered. (B) Expression of *chil-27p*::*GFP* is regulated by *pals-22* and *pals-25*. Shown are control, *pals-22(icb88)*, *pals-22(icb90)*, *pals-22(icb90) pals-25(icb91)*, and *pals-22(icb90) pals-25(icb92)* animals. The *col-12p*::*mCherry* transgene is constitutively expressed in the epidermis of the early adult. Scale bar, 100 μm. (C) Relative levels of *chil* gene expression. *pals-22/25* regulate *chil* genes that are induced upon infection by oomycete. *FDR<0.01 as calculated by edgeR and limma (see Materials and Methods) indicates the gene is considered to be differentially expressed.

As described above, the RNA-seq analysis of genes regulated by *pals-22/25* indicated that this gene pair controls expression of genes induced by diverse natural pathogens of *C*. *elegans*. Indeed, in a forward genetic screen for *C*. *elegans* genes that regulate expression of the *M*. *humicola*-induced *chil-27p*::*GFP* reporter, we isolated independent loss-of-function alleles of *pals-22* (Fig 8A). These mutant alleles cause constitutive expression of *chil-27p*::*GFP* in the epidermis in the absence of infection (Fig 8B). RNAi against *pals-22* also led to constitutive GFP expression (S5 Fig) in a manner that is indistinguishable from that observed upon infection with *M*. *humicola*. These results indicate that *pals-22* acts as a negative regulator of *chil-27* expression in the epidermis.

We then used a *pals-22; chil-27p*::*GFP* strain for a suppressor screen, analogous to the one described earlier for suppressors of GFP expression in *pals-22; pals-5p*::*GFP* (Fig 1). Interestingly, in this new screen we isolated two new alleles of *pals-25* which fully suppress the constitutive expression of *chil-27p*::*GFP* seen in *pals-22* mutants (Fig 8A and 8B), indicating that wild-type *pals-25* acts as a positive regulator of *chil-27* expression. These observations are consistent with our RNA-seq differential gene expression analysis, which determined that *chil-27* is induced in a *pals-22* mutant background and that *pals-25* is required for this induction (Fig 8C). Therefore, *pals-22/25* act as a switch not only for genes induced in the intestine by natural intestinal pathogens, but also for genes induced in the epidermis by natural epidermal pathogens of *C*. *elegans*.

## Discussion

**Fig 9:**
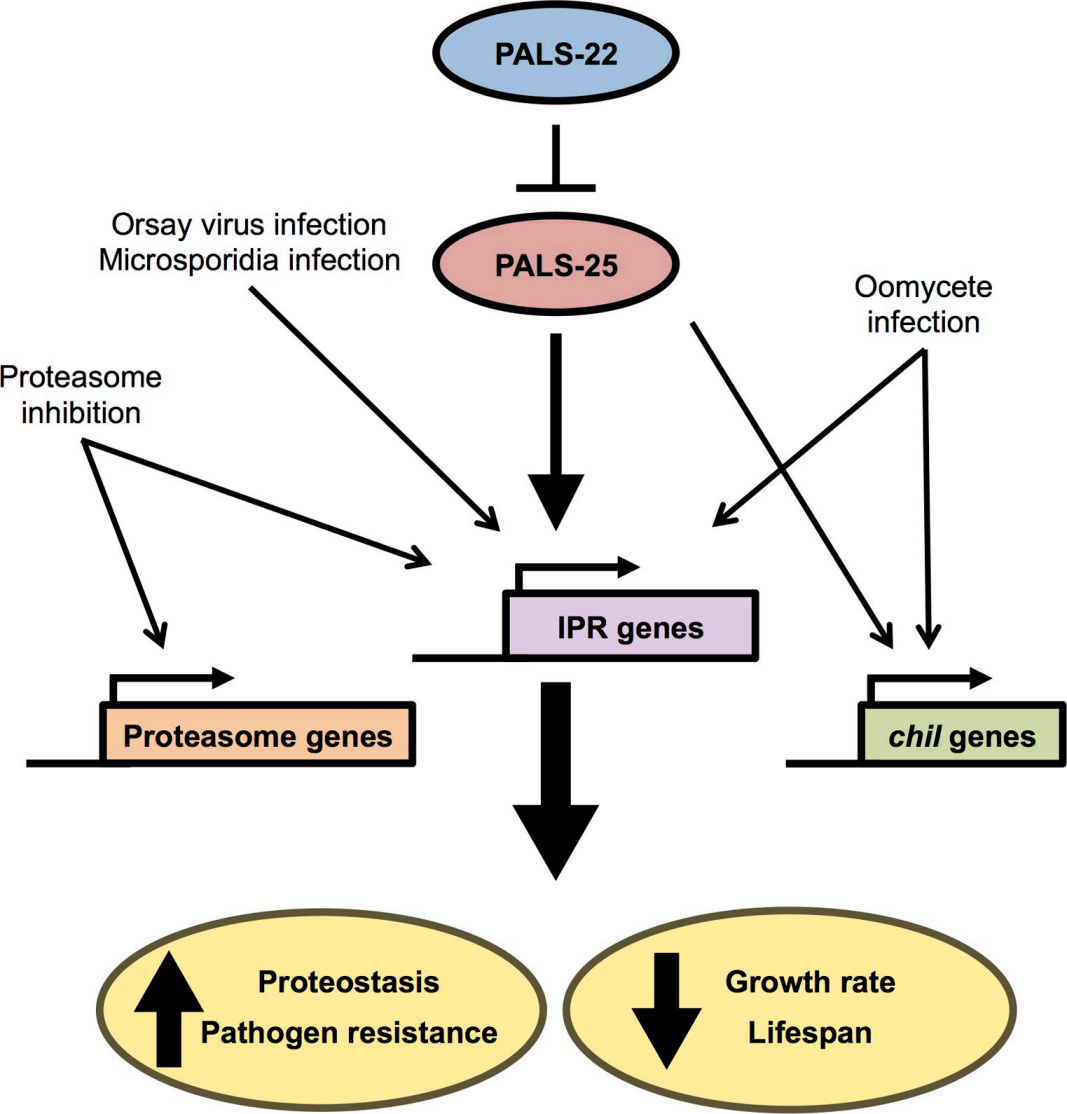
Model for *pals-22/pals-25* regulation of response to natural pathogens of *C*. *elegans*.

In many organisms, there is a balance between growth and pathogen resistance. In particular, many studies in plants have indicated that genetic immunity to disease comes at a cost to the yield of crops [26]. Here we define a program in *C*. *elegans* that controls a balance between organismal growth with resistance to natural pathogens, which is regulated by the *pals-22/25* species-specific gene pair. These genes act as a switch between a 'defense program' of enhanced resistance against diverse natural pathogens like microsporidia and virus, improved tolerance of proteotoxic stress and increased defense against exogenous RNA [8], and a 'growth program' of normal development and lifespan (Fig 9). We call this physiological defense program the "IPR" and it appears to be distinct from other canonical stress response pathways in *C*. *elegans*, including the p38 MAP kinase pathway, the insulin-signaling pathway, and the heat shock response, among others [6]. Our previous analyses indicated that ubiquitin ligases may play a role in executing the IPR program, as the cullin/CUL-6 ubiquitin ligase subunit is required for the enhanced proteostasis capacity of *pals-22* mutants [6].

Our analysis indicates that *pals-22* is genetically upstream of *pals-25*: *pals-25* mutations have effects if *pals-22* is mutated, but there is not an obvious effect of *pals-25* mutations in a wild-type background. Interestingly, the *pals-22* gene is also upstream of the *pals-25* gene in an operon and the two genes are transcribed together. Whether the operon structure of *pals-22/25* contributes to their genetic interactions is unknown, but neither appeared to have a substantial effect on mRNA expression of the other. Therefore, we favor a model whereby the epistatic interaction between these two genes is due to an interaction between their protein products, rather than because one gene affects the transcription of the other. Interestingly, *pals-22/25* are part of a species-specific expansion of the *pals* gene family in *C*. *elegans*, and their genomic location is near F-box-containing genes that are among the most rapidly diversifying genes in the *C*. *elegans* genome. It has been proposed that these genes, as well as other species-specific gene family expansions, are involved in immune defense in *C*. *elegans* [27–30].

*pals-22* mutants are highly resistant to the microsporidian pathogen *N*. *parisii*, which is the most common parasite found in wild-caught *C*. *elegans* [18, 31]. Little is known about innate immune pathways that provide defense against *N*. *parisii*. Canonical immune pathways in *C*. *elegans* like the p38 MAP kinase pathway provide defense against most pathogens tested in *C*. *elegans* but do not provide defense against *N*. *parisii* [12]. The mechanism by which *pals-22/25* regulate resistance to *N*. *parisii* is not clear. Our RNA-seq analysis demonstrates that *pals-22/25* affect expression of hundreds of genes in the genome. In particular, most of the genes induced by the natural intracellular pathogens Orsay virus and *N*. *parisii* are controlled by *pals-22* and *pals-25*, although the function of these IPR genes in defense is unknown. Interestingly, we found that *pals-22/25* regulate expression of genes induced not only by natural intestinal pathogens but also of genes induced by natural epidermal pathogens, such as the oomycete species *M*. *humicola*. *M*. *humicola* induces expression of *chil-*gene family, and genetic analysis shows these genes promote defense against *M*. *humicola* [24]. Notably, we identified *pals-22/25* in independent forward genetic screens for regulators of *chil-27* and found that they regulate expression of this defense gene in the epidermis. Thus, *pals-22/25* regulate expression of genes induced by diverse natural pathogens.

While *pals-25* is required to activate IPR gene expression in a *pals-22* mutant background, it is not required for activation of IPR gene expression in response to *N*. *parisii* infection or proteasomal stress. Therefore, *pals-22/25* may not mediate detection of these triggers, although they might mediate detection and be redundant with other factors. Intriguingly, the *pals-22/25* gene pair share evolutionary and phenotypic features with plant R gene pairs, which serve as sensors for virulence factors delivered into host cells by co-evolved plant pathogens. For example, the *Arabidopsis thaliana* gene pair RRS1 and RPS4 are species-specific, share the same promoter, and direct opposite outcomes, with RRS1 inhibiting and RPS4 promoting 'effector-triggered immunity' against natural pathogens [32, 33]. Similarly, *pals-22* and *pals-25* are species-specific, appear to be in an operon together, and direct opposite physiological outcomes including defense against natural pathogens. RRS1 and RPS4 proteins directly bind to each other, and RRS1 normally inhibit RPS4 function until detection of bacterial virulence factors, at which point RRS1 inhibition is relieved and RPS4 is free to promote pathogen defense, although the steps downstream of RRS1/RPS4 are poorly understood. Although the *pals* genes do not share sequence similarity with the R genes, in this analogy PALS-22 would inhibit PALS-25 and serve as the 'tripwire' to detect virulence factors from natural pathogens and free PALS-25 to promote the IPR defense program. While this model is attractive, it is purely speculative as we currently have no direct evidence that PALS-22 detects virulence factors. Identification of such hypothetical virulence factors would be the focus of future studies.

The molecular events by which *C*. *elegans* detects infection are poorly understood, although nematodes do appear to use a form of effector-triggered immunity or ‘surveillance immunity’. Studies with several distinct pathogens have indicated that *C*. *elegans* induces defense gene expression in response to perturbation of core processes like translation and the ubiquitin-proteasome system [34]. For example, studies with *P*. *aeruginosa* demonstrated that *C*. *elegans* detects the presence of the translation-blocking Exotoxin A through its effects on host translation, not through detection of the shape of the toxin [35, 36]. In addition to this mode of detection, *C*. *elegans* may also detect specific molecular signatures like canonical Pathogen-Associated Molecular Patterns (PAMPs). In all likelihood, several types of pathogen detection are used by *C*. *elegans*. Surprisingly however, there have been no direct PAMP ligand/receptor interactions demonstrated for pattern recognition receptors (PRR) in the worm, although there has been a Damage-Associated Molecular Pattern (DAMP)/G-protein-coupled receptor interaction demonstrated to be critical for response to *Drechmeria* infection [37]. Indeed, *C*. *elegans* lacks many PRR signaling pathways that are well described in flies and mammals. For example, the *C*. *elegans* single Toll-like receptor *tol-1* does not act canonically and worms appear to have lost its downstream transcription factor NFkB, which is critical for innate immunity in flies and mammals [38]. Perhaps conservation of immune genes is only reserved for defense against rare, ‘non-natural’ pathogens, because genes that are important for immunity are subject to attack and inhibition by microbes [39]. Thus, immune genes that provide defense against natural pathogens from the recent evolutionary past will not be broadly conserved but rather will be species-specific, like rapidly evolving R genes in plants. While R genes have been shown to encode proteins that detect virulence factors secreted into host cells by co-evolved plant pathogens, the mechanism by which they activate downstream immune signaling is unclear. We propose that the IPR physiological program regulated by the *pals-22/25* antagonistic paralogs in *C*. *elegans* could be analogous to effector-triggered immunity regulated by opposing R gene pairs like RRS1/RPS4 used in plants for resistance against co-evolved pathogens.

Interestingly, an example of vertebrate-specific antagonistic paralogs has recently been described to play a role in regulating nonsense-mediated RNA decay (NMD) [40]. These studies provide a potential explanation to the long-standing question of how gene duplications are retained, when they are presumably redundant immediately following gene duplication. Specifically, this model predicts that gene duplication events can be rapidly retained if the proteins made from these genes are involved in protein-protein interactions. With just one non-synonymous nucleotide change that switches a wild-type copy to become dominant negative within a multimeric signaling complex, a gene duplication event can be selected for and retained in the heterozygote state–i.e. in one generation. Perhaps in this way, new genes can be born and survive, when gene pairs can evolve to direct opposing functions like the Upf3a/3b paralogs in NMD, and the RRS1/RPS4 and *pals-22/25* paralogs in immunity/growth.

## Methods

### Strains

*C*. *elegans* were maintained at 20°C on Nematode Growth Media (NGM) plates seeded with Streptomycin-resistant *E*. *coli* OP50-1 bacteria according to standard methods [41]. We used N2 wild-type animals. Mutant or transgenic strains were backcrossed at least three times. See S1 Table for a list of all strains used in this study.

### EMS screens and cloning of alleles

*pals-22* mutant worms (either the *jy1* or *jy3* allele) carrying the *jyIs8[pals-5p*::*GFP*, *myo-2p*::*mCherry]* transgene were mutagenized with ethyl methane sulfonate (EMS) (Sigma) using standard procedures as described [42]. L4 stage P0 worms were incubated in 47 mM EMS for 4 hours at 20°C. Worms were screened in the F2 generation for decreased expression of GFP using the COPAS Biosort machine (Union Biometrica). Complementation tests were carried out by generating worms heterozygous for two mutant alleles and scoring *pals-5p*::*GFP* fluorescence. For whole-genome sequencing analysis of mutants, genomic DNA was prepared using a Puregene Core kit (Qiagen) and 20X sequencing coverage was obtained. We identified only one gene (*pals-25*) on LGIII containing variants predicted to alter function in both mutants sequenced (*jy9* and *jy100*). Additional *pals-25* alleles were identified by Sanger sequencing. Screens carried out in the strains with the *icbIs4[chil-27p*::*GFP*, *col-12p*::*mCherry]* transgene were performed in a similar manner except that we used 24 mM EMS to recover the *pals-22* alleles (*icb88*, *icb90*) and 17 mM EMS for the *pals-22(icb90)* suppressor screen and that in both cases we selected F2 animals manually using a Zeiss Axio ZoomV16 dissecting scope. The two *pals-22* alleles (*icb88*, *icb90*) were identified by whole genome sequencing of GFP positive F2 recombinants after crossing to the polymorphic isolate CB4856 as previously described [43] whereas the two *pals-25* alleles were found by direct sequencing of the mutant strains. The *pals-22(icb89)* allele was identified by Sanger sequencing. See S1 Table for a list of all mutations identified.

### CRISPR-Cas9 deletion of *pals-22/25* and *pals-25* genes

The co-conversion strategy [44] was used to generate *pals-22/25* and *pals-25* deletion mutants with *dpy-10* as the selection marker. CRISPR RNAs (crRNAs) were designed to target the *pals-22* upstream region (CCGCTAGTTTTCAGACCTCT), the *pals-25* upstream region (AACCCTGTAAAGCTACAGTA), and the *pals-25* downstream region (GCCCTTGTATGCTGATCCTC). These crRNAs were synthesized (IDT), annealed with tracrRNA (IDT), mixed with purified Cas9 protein (QB3 MacroLab), and then injected into gonads of N2 worms. Dumpy or roller F1 animals were selected and then genotyped for deletions in the *pals-22/25* or *pals-25* region. The *jy79*, *jy80*, *jy81*, and *jy82* mutants were backcrossed to N2 three times before characterization.

### RNA interference

RNA interference was performed using the feeding method. Overnight cultures of RNAi clones in the HT115 bacterial strain were seeded onto NGM plates supplemented with 5mM IPTG and 1mM carbenicillin and incubated at 25°C for 1 day. Eggs from bleached parents or synchronized L1 stage animals were fed RNAi until the L4 stage at 20°C. For all RNAi experiments an *unc-22* clone leading to twitching animals was used as a positive control to test the efficacy of the RNAi plates. The *pals-22* RNAi clone (from the Ahringer RNAi library) was verified by sequencing and was effective in inducing *pals-5p*::*GFP* expression. The *pals-25* RNAi clone was made with PCR and includes 1079 base pairs spanning the second, third, and fourth exons of *pals-25*. This sequence was amplified from N2 genomic DNA, cloned into the L4440 RNAi vector, and then transformed into HT115 bacteria for feeding RNAi experiments.

### Quantitative RT-PCR

Endogenous mRNA expression changes were measured with qRT-PCR as previously described [6]. Synchronized L1 worms were grown on NGM plates at 20°C to the L4 stage and then collected in TriReagent (Molecular Research Center, Inc.) for RNA extraction. For *N*. *parisii* infection, 7 x 10^6^ spores were added to plates with L4 stage worms and then incubated at 25°C for 4 hours before RNA isolation. Bortezomib (or an equivalent amount of DMSO) was added to L4 stage worms for a final concentration of 20 μM; plates were then incubated at 20°C for 4 hours before RNA isolation. At least two independent biological replicates were measured for each condition, and each biological replicate was measured in duplicate and normalized to the *snb-1* control gene, which did not change upon conditions tested. The Pffafl method was used for quantifying data [45].

### Heat shock assay

Worms were grown on standard NGM plates until the L4 stage at 20°C and then shifted to 37°C for two hours. Following heat shock, plates were laid in a single layer on the bench top for 30 minutes to recover, and then moved to a 20°C incubator overnight (dx.doi.org/10.17504/protocols.io.v6re9d6). Worms were scored in a blinded manner for survival 24 hours after heat shock; animals not pumping or responding to touch were scored as dead. Three plates were assayed for each strain in each replicate, with at least 30 worms per plate, and three independent assays were performed.

### GFP fluorescence measurement

Synchronized L1 stage animals were grown at 20°C to the L4 stage. The COPAS Biosort machine (Union Biometrica) was used to measure the time of flight (as a measure of length) and fluorescence of individual worms. At least 100 worms were measured for each strain, and all experiments were performed in biological triplicate.

### Lifespan

L4 stage worms were transferred to 6 cm NGM plates seeded with OP50-1 bacteria and incubated at 25°C. Worms were scored every day, and animals that did not respond to touch were scored as dead. Animals that died from internal hatching or crawled off the plate were censored. Worms were transferred to new plates every day throughout the reproductive period. Three plates were assayed for each strain in each replicate, with 40 worms per plate.

### Microscopy

Worms were anesthetized with 10 μM levamisole in M9 buffer and mounted on 2% agarose pads for imaging. Images in S1B and S1C Fig were captured with a Zeiss LSM700 confocal microscope. All other *C*. *elegans* images were captured with a Zeiss AxioImager M1 or Axio Zoom V16.

### *N*. *parisii* and Orsay virus infection assays

*N*. *parisii* spores were prepared as previously described [46], and Orsay virions were prepared as described previously [9]. For pathogen load analysis (dx.doi.org/10.17504/protocols.io.waiface), synchronized L1 worms were plated with a mixture of OP50 bacteria and 5 x 10^5^ *N*. *parisii* spores or a 1:20 dilution of Orsay virus filtrate, and then incubated at 25°C for either 30 hours (*N*. *parisii*) or 18 hours (Orsay virus) before fixing with paraformaldehyde. Fixed worms were stained with individual FISH probes conjugated to the red Cal Fluor 610 dye (Biosearch Technologies) targeting either *N*. *parisii* ribosomal RNA or Orsay virus RNA. *N*. *parisii* pathogen load was measured with the COPAS Biosort machine (Union Biometrica). Orsay virus infection was assayed visually using the 10x objective on a Zeiss AxioImager M1 microscope. In feeding measurement assays (dx.doi.org/10.17504/protocols.io.v6ne9de), plates were set up as for pathogen infection with the addition of fluorescent beads (Fluoresbrite Polychromatic Red Microspheres, Polysciences Inc.). Worms were fixed in paraformaldehyde after 30 minutes and red fluorescence signal was measured with the COPAS Biosort machine (Union Biometrica).

### *P*. *aeruginosa* pathogen load

Overnight cultures of a *P*. *aeruginosa* PA14-dsRed strain [47] were seeded onto SK plates with 50 μg/ml ampicillin, and then incubated at 37°C for 24 hours followed by 25°C for 24 hours. Worms at the L4 stage were washed onto the PA14-dsRed plates, incubated at 25°C for 16 hours, and then assayed with a COPAS Biosort machine (Union Biometrica) for the amount of red fluorescence inside each animal.

### RNA-seq sample preparation

Synchronized L1 stage worms were grown on 10 cm NGM plates seeded with OP50-1 *E*. *coli* at 20°C until worms had reached the L4 stage. N2, *pals-22(jy3)*, and *pals-22(jy3) pals-25(jy9)* strains were then shifted to 25°C for 4 hours before harvesting for RNA extraction. Bortezomib (or an equivalent amount of DMSO) was added to plates with L4 stage N2 worms for a final concentation of 20 μM; plates were then incubated at 20°C for 4 hours before RNA isolation. RNA was isolated with TriReagent purification, followed by RNeasy column cleanup (Qiagen), as described [48]. RNA quality was assessed by Tapestation analysis at the Institute for Genomic Medicine (IGM) at UC San Diego. Paired-end sequencing libraries were then constructed with the TruSeq Stranded mRNA method (Illumina), followed by sequencing on HiSeq4000 machine (Illumina). RNA-seq reads were uploaded to the NCBI GEO database with Accession number GSE118400.

### RNA-seq analysis

Sequencing reads were aligned to WormBase release WS235 using Bowtie 2 [49], and transcript abundance was estimated using RSEM [50]. Differential expression analysis was performed in RStudio (v1.1.453) [51] using R (v3.50) [52] and Bioconductor (v3.7) [53] packages. As outlined in the RNAseq123 vignette [54], data was imported, filtered and normalized using edgeR [55], and linear modeling and differential expression analysis was performed using limma [56]. An FDR [57] cutoff of <0.01 was used to define differentially expressed genes; no fold-change criteria was used. Lists of upregulated genes used for comparisons were exported and further sanitized to remove dead genes and update WBGeneIDs to WormBase release WS263. Pseudogenes were annotated using a list obtained from WormBase release WS262.

### Functional enrichments analyses

Functional analysis was performed using Gene Set Enrichment Analysis (GSEA) v3.0 software [58, 59]. Normalized RNA-seq expression data were converted into a GSEA-compatible filetype and ranked using the signal-to-noise metric with 1,000 permutations. Gene sets from other studies were converted to WBGeneIDs according to WormBase release WS263. Independent analyses were performed for each of three comparisons: untreated *pals-22(jy3)* versus untreated N2 animals; untreated *pals-22(jy3)* versus untreated *pals-22(jy3) pals-25(jy9)* animals; bortezomib treated N2 versus DMSO vehicle control treated N2. Results were graphed based on their NES-value using GraphPad Prism 7 (GraphPad Software, La Jolla, CA).

Gene sets comparison lists were generated using WormMine and formatted in Excel. Representation factors and significance of overlaps were calculated using a hypergeometric test implemented at (nemates.org). For “total number of genes” input we use the most conservative number 11,387, which corresponds to the size of our RNA-seq dataset following the filtering of low-count and undetected genes.

### Reciprocal BLAST search for *pals-22/25* in *C*. *inopinata*

Amino acid sequences for PALS-22 and PALS-25 were searched against the *C*. *inopinata* (*C*. sp34 NK74SC) proteome using BLASTP with default settings (E-value<1.0E-10) (http://blast.caenorhabditis.org). PALS-22 returns no hit using default settings while PALS-25 returns a single hit, CSP34.Sp34_50171800.t1. When this is searched against *C*. *elegans* with BLASTP it returns PALS-39 as the top hit, while PALS-25 is the seventh hit. When *C*. *elegans* PALS-39 is searched against *C*. *inopinata*, CSP34.Sp34_50171800.t1 is returned as the top hit. Therefore, this protein appears to be a PALS-39 ortholog.

## Supporting information

S1 Fig *pals-25* RNAi suppresses increased *pals-5p::GFP* expression in *pals-22* mutants and PALS-25::GFP is expressed broadly. (A) Wild-type or *pals-22* mutant animals carrying the *pals-5p*::*GFP* transgene, treated with either L4440 RNAi control or *pals-25* RNAi. Green is *pals-5p*::*GFP*, red is *myo-2p*::*mCherry* expression in the pharynx as a marker for presence of the transgene. Images are overlays of green, red, and Nomarski channels and were taken with the same camera exposure for all. Scale bar, 100 μm. (B,C) Confocal fluorescence images of adult animals carrying a fosmid transgene expressing PALS-25::GFP from the endogenous promoter. Animals were treated with either (B) L4440 RNAi control or (C) *pals-22* RNAi. Scale bar, 50 μm.
(TIF)

S2 Fig *pals-25* mutation suppresses the lifespan and thermotolerance phenotypes of *pals-22* mutants. (A,B) Lifespan of wild type, *pals-22(jy3)*, and *pals-22(jy3) pals-25(jy9)* animals. Assays were performed with 40 animals per plate, and three plates per strain per experiment. p-value for *pals-22(jy3)* compared to *pals-22(jy3) pals-25(jy9)* is <0.0001 using the Log-rank test. (C,D) Survival of animals after 2 hour heat shock treatment at 37°C followed by 24 hours at 20°C. Strains were tested in triplicate, with at least 30 animals per plate. Mean fraction alive indicates the average survival among the triplicates, errors bars are SD. ** p < 0.01, * p < 0.05.
(TIF)

S3 Fig Mutation of *pals-22* or *pals-25* does not affect feeding rates of animals in pathogen infection assays. (A-C) Quantification of fluorescent bead accumulation in wild-type, *pals-22*, *pals-22 pals-25*, *pals-25*, and *eat-2* mutant animals. Beads were mixed with OP50-1 bacteria and either (A, C) *N*. *parisii* spores or (B) Orsay virus and fed to worms as in infection assays. Worms were fixed in paraformaldehyde after 30 minutes of feeding, and fluorescence of accumulated beads in each animal was measured using a COPAS Biosort machine to measure the mean red signal and length of individual animals, indicated by red dots. Mean signal of the population is indicated by black bars, with error bars as SD. Graph is a compilation of three independent replicates, with at least 100 animals analyzed in each replicate. Statistical analysis was performed using one-way ANOVA. *** p < 0.001, ns, not significant.
(TIFF)

S4 Fig qRT-PCR analysis of *pals-22* and *pals-25* mRNA expression in *pals-25(jy81)* mutants and wild-type animals. qRT-PCR measurement of *pals-22* and *pals-25* gene expression, shown as the fold change relative to wild-type. Results shown are the average of three independent biological replicates and error bars are SD. ns, not significant with Student’s t-test.
(TIFF)

S5 Fig Induction of *chil-27p::GFP* expression seen after *pals-22* RNAi treatment. Animals treated with either L4440 RNAi control, *pals-22* RNAi, or exposed to *M*. *humicola*. The *col-12p*::*mCherry* transgene is constitutively expressed in the epidermis at the early adult stage. Scale bar, 100 μm.
(TIF)

S1 Table Lists of strains and mutations. (XLSX)

S2 Table RNA-seq statistics. (XLSX)

S3 Table FPKM values for all genes in data set. (XLSX)

S4 Table Differentially expressed genes, as determined by edgeR and limma. (XLSX)

S5 Table Gene set overlaps. (XLSX)

S6 Table Gene sets used for GSEA and their sources. (XLSX)

S7 Table Detailed GSEA results. (XLSX)

S8 Table Human orthology analysis. (XLSX)

## References

[1] Pearson JC, Lemons D, McGinnis W. Modulating Hox gene functions during animal body patterning. Nature Reviews Genetics. 2005;6(12):893 10.1038/nrg1726 16341070

[2] Ross BD, Rosin L, Thomae AW, Hiatt MA, Vermaak D, de la Cruz AFA, et al Stepwise evolution of essential centromere function in a Drosophila neogene. Science. 2013;340(6137):1211–4. 10.1126/science.1234393 23744945 PMC4119826

[3] Joshi RK, Nayak S. Perspectives of genomic diversification and molecular recombination towards R-gene evolution in plants. Physiology and Molecular Biology of Plants. 2013;19(1):1–9. 10.1007/s12298-012-0138-2 24381433 PMC3550690

[4] von Moltke J, Ayres JS, Kofoed EM, Chavarría-Smith J, Vance RE. Recognition of bacteria by inflammasomes. Annual review of immunology. 2013;31:73–106. 10.1146/annurev-immunol-032712-095944 23215645

[5] Li X, Kapos P, Zhang Y. NLRs in plants. Current opinion in immunology. 2015;32:114–21. 10.1016/j.coi.2015.01.014 25667191

[6] Reddy KC, Dror T, Sowa JN, Panek J, Chen K, Lim ES, et al An intracellular pathogen response pathway promotes proteostasis in C. elegans. Current Biology. 2017;27(22):3544–53. e5. 10.1016/j.cub.2017.10.009 29103937 PMC5698132

[7] Hentati A, Bejaoui K, Pericak-Vance MA, Hentati F, Speer MC, Hung W-Y, et al Linkage of recessive familial amyotrophic lateral sclerosis to chromosome 2q33–q35. Nature genetics. 1994;7(3):425 10.1038/ng0794-425 7920663

[8] Leyva-Díaz E, Stefanakis N, Carrera I, Glenwinkel L, Wang G, Driscoll M, et al Silencing of repetitive DNA is controlled by a member of an unusual Caenorhabditis elegans gene family. Genetics. 2017;207(2):529–45. 10.1534/genetics.117.300134 28801529 PMC5629321

[9] Bakowski MA, Desjardins CA, Smelkinson MG, Dunbar TA, Lopez-Moyado IF, Rifkin SA, et al Ubiquitin-mediated response to microsporidia and virus infection in C. elegans. PLoS pathogens. 2014;10(6):e1004200 10.1371/journal.ppat.1004200 24945527 PMC4063957

[10] Zuryn S, Jarriault S, editors. Deep sequencing strategies for mapping and identifying mutations from genetic screens. Worm. 2013; 2(3):e25081. 24778934 10.4161/worm.25081 PMC3875646

[11] Sarov M, Murray JI, Schanze K, Pozniakovski A, Niu W, Angermann K, et al A genome-scale resource for in vivo tag-based protein function exploration in C. elegans. Cell. 2012;150(4):855–66. 10.1016/j.cell.2012.08.001 22901814 PMC3979301

[12] Troemel ER, Félix M-A, Whiteman NK, Barrière A, Ausubel FM. Microsporidia are natural intracellular parasites of the nematode Caenorhabditis elegans. PLoS biology. 2008;6(12):e309. 10.1371/journal.pbio.0060309 PMC2596862 19071962

[13] Hsu A-L, Murphy CT, Kenyon C. Regulation of aging and age-related disease by DAF-16 and heat-shock factor. Science. 2003;300(5622):1142–5. 10.1126/science.1083701 12750521

[14] Singh V, Aballay A. Heat-shock transcription factor (HSF)-1 pathway required for Caenorhabditis elegans immunity. Proceedings of the National Academy of Sciences. 2006;103(35):13092–7. 10.1073/pnas.0604050103 PMC1559758 16916933

[15] Vihervaara A, Sistonen L. HSF1 at a glance. Journal of Cell Science. 2014;127:261–6. 24421309 10.1242/jcs.132605

[16] Raizen DM, Lee R, Avery L. Interacting genes required for pharyngeal excitation by motor neuron MC in Caenorhabditis elegans. Genetics. 1995;141(4):1365–82. 8601480 10.1093/genetics/141.4.1365 PMC1206873

[17] Félix M-A, Ashe A, Piffaretti J, Wu G, Nuez I, Bélicard T, et al Natural and experimental infection of Caenorhabditis nematodes by novel viruses related to nodaviruses. PLoS biology. 2011;9(1):e1000586 10.1371/journal.pbio.1000586 21283608 PMC3026760

[18] Schulenburg H, Félix M-A. The natural biotic environment of Caenorhabditis elegans. Genetics. 2017;206(1):55–86. 10.1534/genetics.116.195511 28476862 PMC5419493

[19] Kim D, Ewbank J. Signaling in the Immune Response. 2018:1–35. WormBook [Internet] http://www.wormbook.org.

[20] Pukkila-Worley R, Ausubel FM. Immune defense mechanisms in the Caenorhabditis elegans intestinal epithelium. Current opinion in immunology. 2012;24(1):3–9. 10.1016/j.coi.2011.10.004 22236697 PMC3660727

[21] Oliveira RP, Abate JP, Dilks K, Landis J, Ashraf J, Murphy CT, et al Condition‐adapted stress and longevity gene regulation by Caenorhabditis elegans SKN‐1/Nrf. Aging cell. 2009;8(5):524–41. 10.1111/j.1474-9726.2009.00501.x 19575768 PMC2776707

[22] Li J, Chauve L, Phelps G, Brielmann RM, Morimoto RI. E2F coregulates an essential HSF developmental program that is distinct from the heat-shock response. Genes Dev. 2016;30(18):2062–75. Epub 2016/11/01. 10.1101/gad.283317.116 27688402 PMC5066613

[23] Irazoqui JE, Troemel ER, Feinbaum RL, Luhachack LG, Cezairliyan BO, Ausubel FM. Distinct pathogenesis and host responses during infection of C. elegans by P. aeruginosa and S. aureus. PLoS pathogens. 2010;6(7):e1000982. 20617181 10.1371/journal.ppat.1000982 PMC2895663

[24] Osman GA, Fasseas MK, Koneru SL, Essmann CL, Kyrou K, Srinivasan MA, et al Natural infection of C. elegans by an oomycete reveals a new pathogen-specific immune response. Current Biology. 2018;28(4):640–8. e5. 10.1016/j.cub.2018.01.029 29398216

[25] Kim W, Underwood RS, Greenwald I, Shaye DD. OrthoList 2: A New Comparative Genomic Analysis of Human and Caenorhabditis elegans Genes. Genetics. 2018;210(2):445–61. 10.1534/genetics.118.301307 30120140 PMC6216590

[26] Ning Y, Liu W, Wang G-L. Balancing Immunity and Yield in Crop Plants. Trends in plant science. 2017;22(12):1069–1079. 29037452 10.1016/j.tplants.2017.09.010

[27] Pujol N, Davis PA, Ewbank JJ. The origin and function of anti-fungal peptides in C. elegans: open questions. Frontiers in immunology. 2012;3:237 10.3389/fimmu.2012.00237 22870075 PMC3409374

[28] Pujol N, Zugasti O, Wong D, Couillault C, Kurz CL, Schulenburg H, et al Anti-fungal innate immunity in C. elegans is enhanced by evolutionary diversification of antimicrobial peptides. PLoS pathogens. 2008;4(7):e1000105 10.1371/journal.ppat.1000105 18636113 PMC2453101

[29] Stewart MK, Clark NL, Merrihew G, Galloway EM, Thomas JH. High genetic diversity in the chemoreceptor superfamily of Caenorhabditis elegans. Genetics. 2005;169(4):1985–96. 10.1534/genetics.104.035329 15520260 PMC1449585

[30] Thomas JH. Concerted evolution of two novel protein families in Caenorhabditis species. Genetics. 2006;172(4):2269–81. 16415360 10.1534/genetics.105.052746 PMC1456376

[31] Zhang G, Sachse M, Prevost M-C, Luallen RJ, Troemel ER, Félix M-A. A large collection of novel nematode-infecting microsporidia and their diverse interactions with Caenorhabditis elegans and other related nematodes. PLoS pathogens. 2016;12(12):e1006093 10.1371/journal.ppat.1006093 27942022 PMC5179134

[32] Le Roux C, Huet G, Jauneau A, Camborde L, Trémousaygue D, Kraut A, et al A receptor pair with an integrated decoy converts pathogen disabling of transcription factors to immunity. Cell. 2015;161(5):1074–88. 10.1016/j.cell.2015.04.025 26000483

[33] Sarris PF, Duxbury Z, Huh SU, Ma Y, Segonzac C, Sklenar J, et al A plant immune receptor detects pathogen effectors that target WRKY transcription factors. Cell. 2015;161(5):1089–100. 10.1016/j.cell.2015.04.024 26000484

[34] Cohen LB, Troemel ER. Microbial pathogenesis and host defense in the nematode C. elegans. Current opinion in microbiology. 2015;23:94–101. 10.1016/j.mib.2014.11.009 25461579 PMC4324121

[35] Dunbar TL, Yan Z, Balla KM, Smelkinson MG, Troemel ER. C. elegans detects pathogen-induced translational inhibition to activate immune signaling. Cell host & microbe. 2012;11(4):375–86. 22520465 10.1016/j.chom.2012.02.008 PMC3334869

[36] McEwan DL, Kirienko NV, Ausubel FM. Host translational inhibition by Pseudomonas aeruginosa Exotoxin A Triggers an immune response in Caenorhabditis elegans. Cell host & microbe. 2012;11(4):364–74. 22520464 10.1016/j.chom.2012.02.007 PMC3334877

[37] Zugasti O, Bose N, Squiban B, Belougne J, Kurz CL, Schroeder FC, et al Activation of a G protein-coupled receptor by its endogenous ligand triggers the Caenorhabditis elegans innate immune response. Nature immunology. 2014;15(9):833 10.1038/ni.2957 25086774 PMC4139443

[38] Irazoqui JE, Urbach JM, Ausubel FM. Evolution of host innate defence: insights from Caenorhabditis elegans and primitive invertebrates. Nature Reviews Immunology. 2010;10(1):47 10.1038/nri2689 20029447 PMC2965059

[39] Maltez VI, Miao EA. Reassessing the evolutionary importance of inflammasomes. The Journal of Immunology. 2016;196(3):956–62. 10.4049/jimmunol.1502060 26802061 PMC4724637

[40] Shum EY, Jones SH, Shao A, Dumdie J, Krause MD, Chan W-K, et al The antagonistic gene paralogs Upf3a and Upf3b govern nonsense-mediated RNA decay. Cell. 2016;165(2):382–95. 10.1016/j.cell.2016.02.046 27040500 PMC4826573

[41] Brenner S. The genetics of Caenorhabditis elegans. Genetics. 1974;77(1):71–94. 4366476 10.1093/genetics/77.1.71 PMC1213120

[42] Kutscher LM, Shaham S. Forward and reverse mutagenesis in C. elegans. WormBook: the online review of C elegans biology. http://www.wormbook.org. 2014:1–26. 10.1895/wormbook.1.167.1 PMC4078664 24449699

[43] Minevich G, Park DS, Blankenberg D, Poole RJ, Hobert O. CloudMap: a cloud-based pipeline for analysis of mutant genome sequences. Genetics. 2012;192(4):1249–69. 10.1534/genetics.112.144204 23051646 PMC3512137

[44] Arribere JA, Bell RT, Fu BX, Artiles KL, Hartman PS, Fire AZ. Efficient marker-free recovery of custom genetic modifications with CRISPR/Cas9 in Caenorhabditis elegans. Genetics. 2014:genetics. 114.169730. 10.1534/genetics.114.169730 PMC4224173 25161212

[45] Pfaffl MW. A new mathematical model for relative quantification in real-time RT–PCR. Nucleic acids research. 2001;29(9):e45–e. 11328886 10.1093/nar/29.9.e45 PMC55695

[46] Balla KM, Andersen EC, Kruglyak L, Troemel ER. A wild C. elegans strain has enhanced epithelial immunity to a natural microsporidian parasite. PLoS pathogens. 2015;11(2):e1004583 10.1371/journal.ppat.1004583 25680197 PMC4334554

[47] Djonović S, Urbach JM, Drenkard E, Bush J, Feinbaum R, Ausubel JL, et al Trehalose biosynthesis promotes Pseudomonas aeruginosa pathogenicity in plants. PLoS pathogens. 2013;9(3):e1003217 10.1371/journal.ppat.1003217 23505373 PMC3591346

[48] Troemel ER, Chu SW, Reinke V, Lee SS, Ausubel FM, Kim DH. p38 MAPK regulates expression of immune response genes and contributes to longevity in C. elegans. PLoS genetics. 2006;2(11):e183 10.1371/journal.pgen.0020183 17096597 PMC1635533

[49] Langmead B, Salzberg SL. Fast gapped-read alignment with Bowtie 2. Nature methods. 2012;9(4):357 10.1038/nmeth.1923 22388286 PMC3322381

[50] Li B, Dewey CN. RSEM: accurate transcript quantification from RNA-Seq data with or without a reference genome. BMC bioinformatics. 2011;12(1):323. 21816040 10.1186/1471-2105-12-323 PMC3163565

[51] Team R. RStudio: integrated development for R RStudio, Inc, Boston, MA URL http://www.rstudio.com. 2015.

[52] Team RC. R: A language and environment for statistical computing 2013 R Foundation for Statistical Computing, Vienna, Austria http://www.R-project.org/.

[53] Gentleman RC, Carey VJ, Bates DM, Bolstad B, Dettling M, Dudoit S, et al Bioconductor: open software development for computational biology and bioinformatics. Genome biology. 2004;5(10):R80 10.1186/gb-2004-5-10-r80 15461798 PMC545600

[54] Law CW, Alhamdoosh M, Su S, Smyth GK, Ritchie ME. RNA-seq analysis is easy as 1-2-3 with limma, Glimma and edgeR. F1000Research. 2016; 5:1408. 10.12688/f1000research.9005.1 PMC4937821 27441086

[55] Robinson MD, McCarthy DJ, Smyth GK. edgeR: a Bioconductor package for differential expression analysis of digital gene expression data. Bioinformatics. 2010;26(1):139–40. 10.1093/bioinformatics/btp616 19910308 PMC2796818

[56] Ritchie ME, Phipson B, Wu D, Hu Y, Law CW, Shi W, et al limma powers differential expression analyses for RNA-sequencing and microarray studies. Nucleic acids research. 2015;43(7):e47–e. 10.1093/nar/gkv007 25605792 PMC4402510

[57] Storey JD, Tibshirani R. Statistical significance for genomewide studies. Proceedings of the National Academy of Sciences. 2003;100(16):9440–5. 10.1073/pnas.1530509100 PMC170937 12883005

[58] Mootha VK, Lindgren CM, Eriksson K-F, Subramanian A, Sihag S, Lehar J, et al PGC-1α-responsive genes involved in oxidative phosphorylation are coordinately downregulated in human diabetes. Nature genetics. 2003;34(3):267 10.1038/ng1180 12808457

[59] Subramanian A, Tamayo P, Mootha VK, Mukherjee S, Ebert BL, Gillette MA, et al Gene set enrichment analysis: a knowledge-based approach for interpreting genome-wide expression profiles. Proceedings of the National Academy of Sciences. 2005;102(43):15545–50. 10.1073/pnas.0506580102 PMC1239896 16199517

