# Supplementary figures and images for: Antagonistic paralogs control a switch between growth and pathogen resistance in C. elegans
Source: PLoS Pathog. 2019 Jan 14;15(1):e1007528. doi: 10.1371/journal.ppat.1007528 (PMC6347328; doi:10.1371/journal.ppat.1007528)

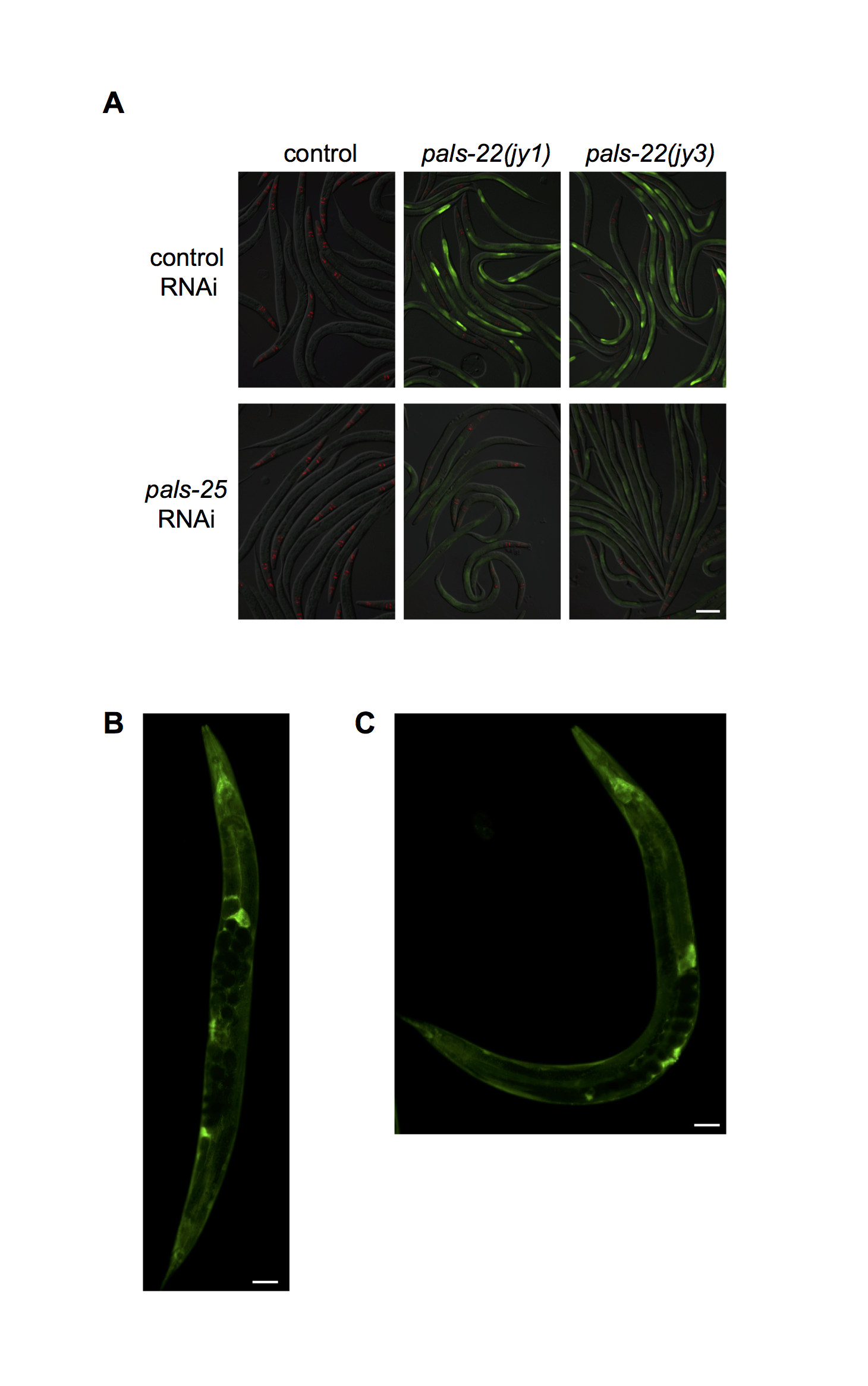

Supplement: S1 Fig — (A) Wild-type or pals-22 mutant animals carrying the pals-5p::GFP transgene, treated with either L4440 RNAi control or pals-25 RNAi. Green is pals-5p::GFP, red is myo-2p::mCherry expression in the pharynx as a marker for presence of the transgene. Images are overlays of green, red, and Nomarski channels and were taken with the same camera exposure for all. Scale bar, 100 μm. (B,C) Confocal fluorescence images of adult animals carrying a fosmid transgene expressing PALS-25::GFP from the endogenous promoter. Animals were treated with either (B) L4440 RNAi control or (C) pals-22 RNAi. Scale bar, 50 μm. (TIF) [file ppat.1007528.s001.tif]

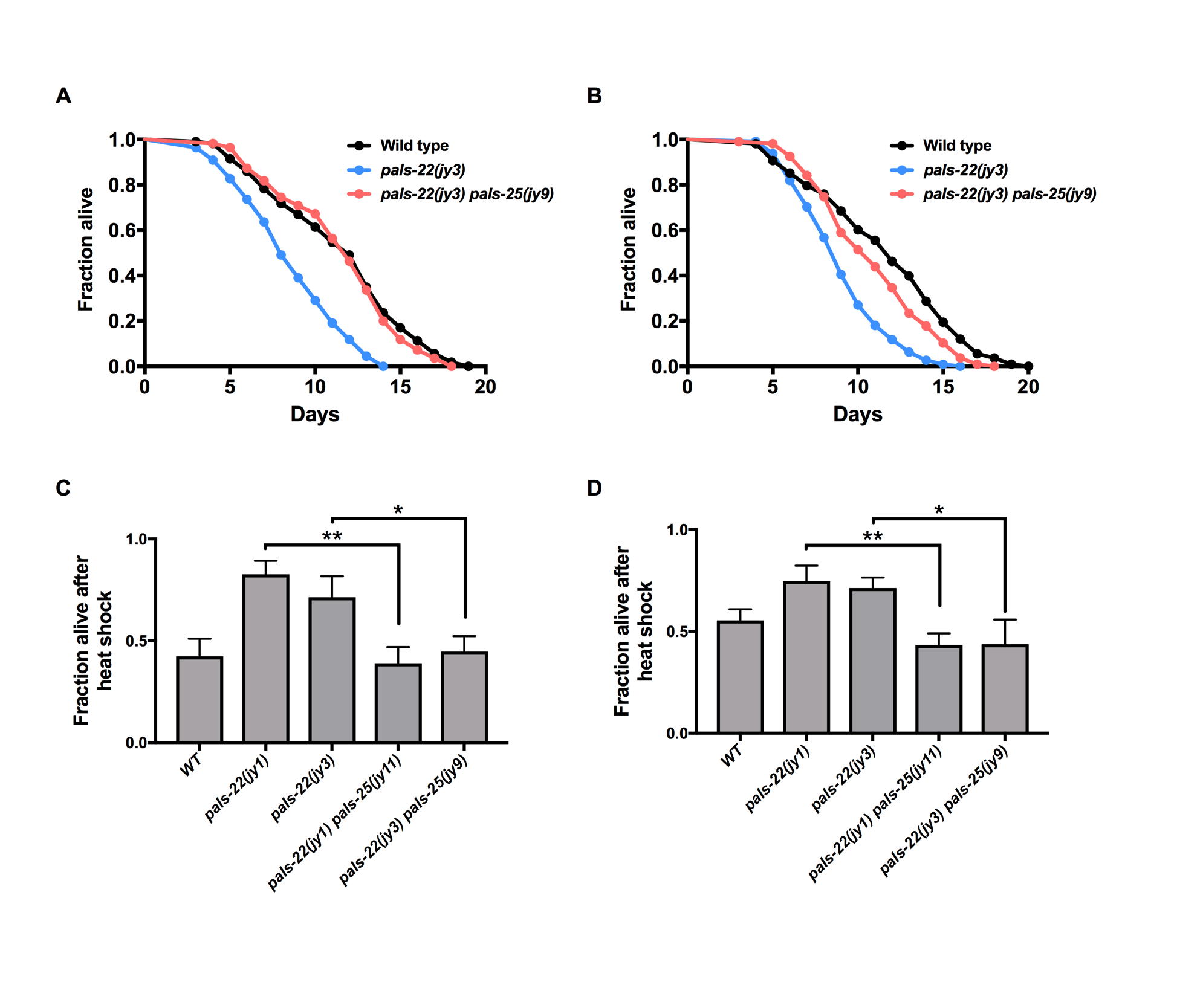

Supplement: S2 Fig — (A,B) Lifespan of wild type, pals-22(jy3), and pals-22(jy3) pals-25(jy9) animals. Assays were performed with 40 animals per plate, and three plates per strain per experiment. p-value for pals-22(jy3) compared to pals-22(jy3) pals-25(jy9) is <0.0001 using the Log-rank test. (C,D) Survival of animals after 2 hour heat shock treatment at 37°C followed by 24 hours at 20°C. Strains were tested in triplicate, with at least 30 animals per plate. Mean fraction alive indicates the average survival among the triplicates, errors bars are SD. ** p < 0.01, * p < 0.05. (TIF) [file ppat.1007528.s002.tif]

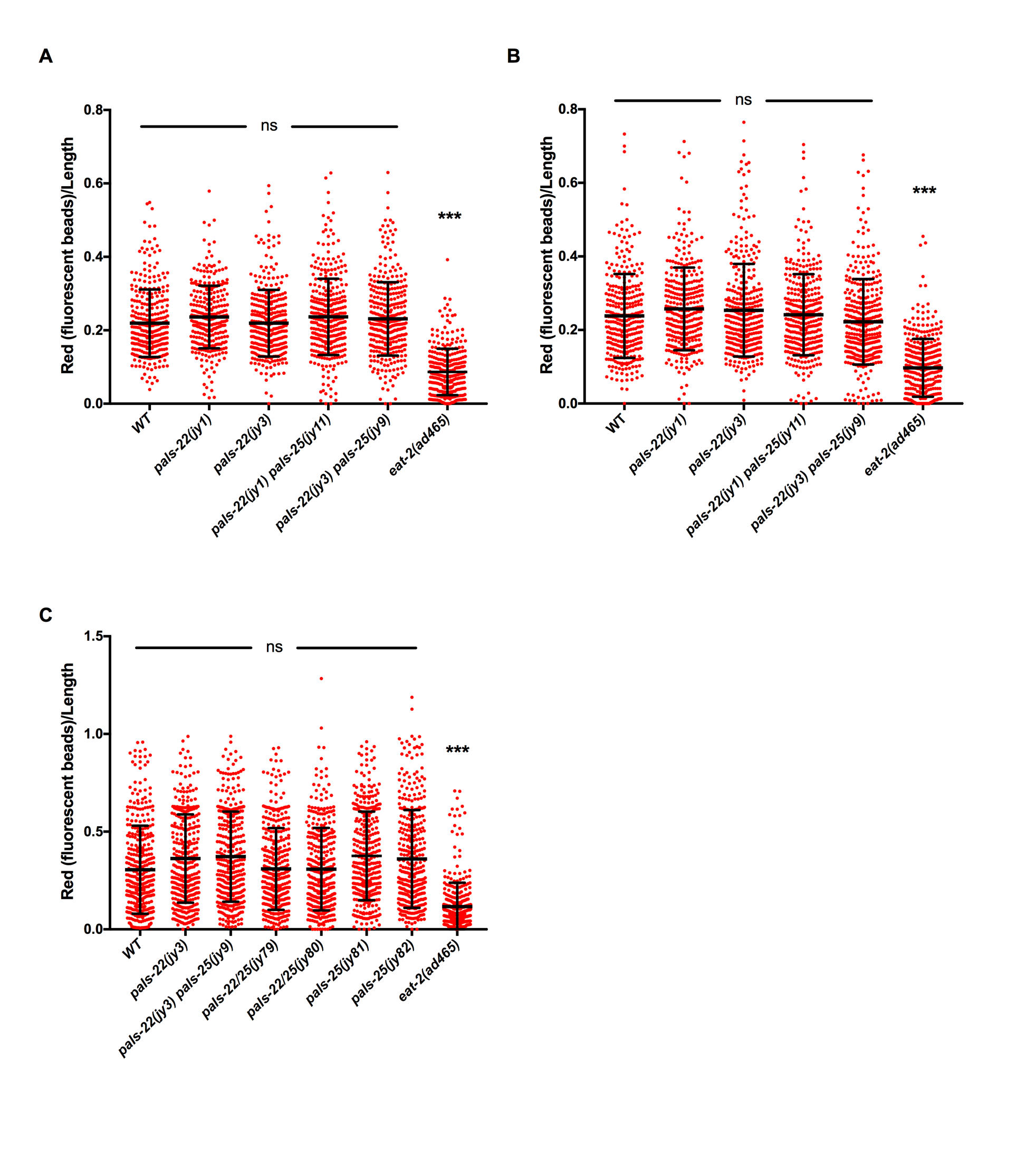

Supplement: S3 Fig — (A-C) Quantification of fluorescent bead accumulation in wild-type, pals-22, pals-22 pals-25, pals-25, and eat-2 mutant animals. Beads were mixed with OP50-1 bacteria and either (A, C) N. parisii spores or (B) Orsay virus and fed to worms as in infection assays. Worms were fixed in paraformaldehyde after 30 minutes of feeding, and fluorescence of accumulated beads in each animal was measured using a COPAS Biosort machine to measure the mean red signal and length of individual animals, indicated by red dots. Mean signal of the population is indicated by black bars, with error bars as SD. Graph is a compilation of three independent replicates, with at least 100 animals analyzed in each replicate. Statistical analysis was performed using one-way ANOVA. *** p < 0.001, ns, not significant. (TIFF) [file ppat.1007528.s003.tiff]

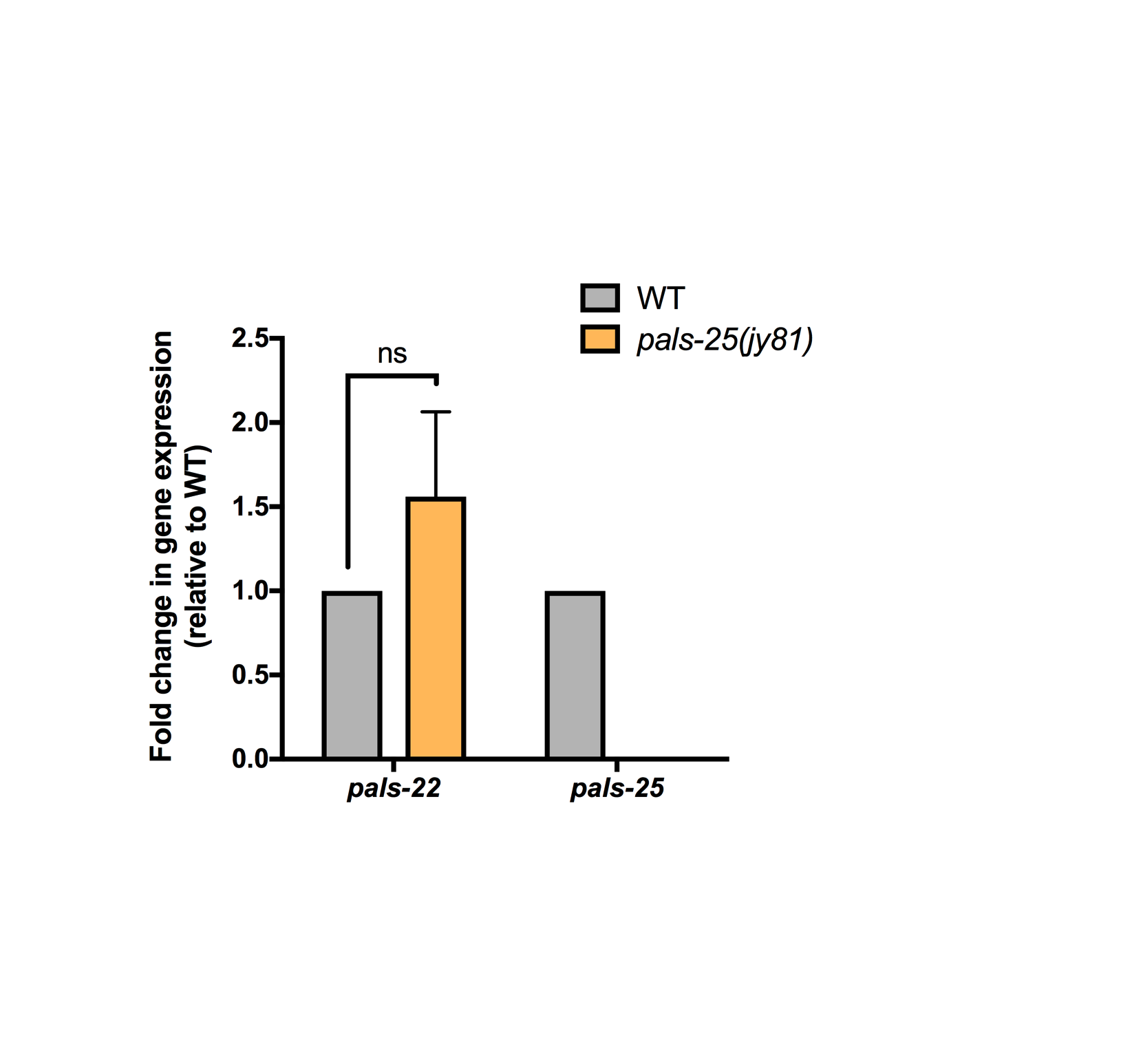

Supplement: S4 Fig — qRT-PCR measurement of pals-22 and pals-25 gene expression, shown as the fold change relative to wild-type. Results shown are the average of three independent biological replicates and error bars are SD. ns, not significant with Student’s t-test. (TIFF) [file ppat.1007528.s004.tiff]

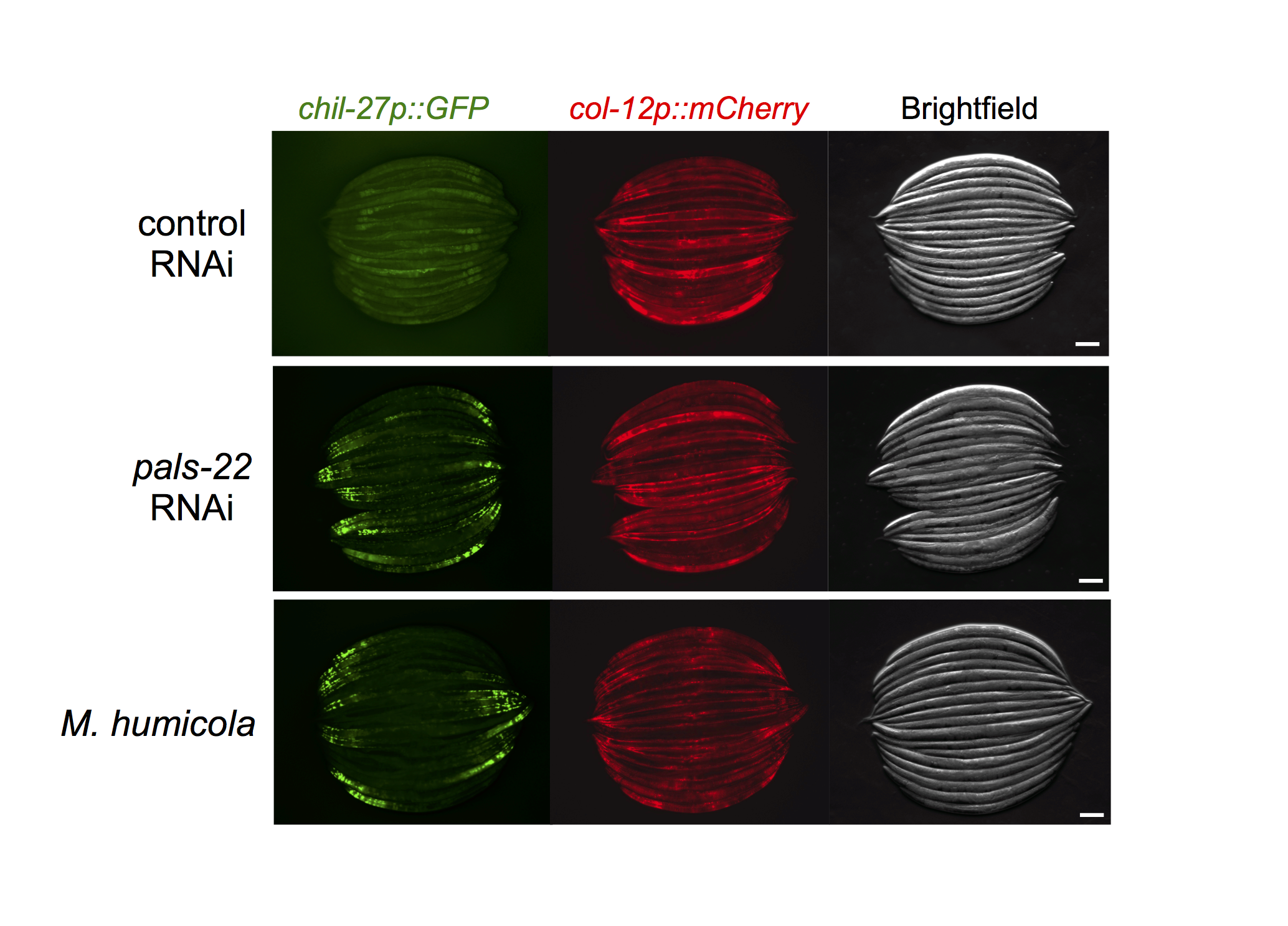

Supplement: S5 Fig — Animals treated with either L4440 RNAi control, pals-22 RNAi, or exposed to M. humicola. The col-12p::mCherry transgene is constitutively expressed in the epidermis at the early adult stage. Scale bar, 100 μm. (TIF) [file ppat.1007528.s005.tif]
